# Homologous desensitization of guanylyl cyclase A, the receptor for atrial natriuretic peptide, is associated with a complex phosphorylation pattern

**DOI:** 10.1111/j.1742-4658.2010.07658.x

**Published:** 2010-06

**Authors:** Juliane Schröter, René P Zahedi, Michael Hartmann, Birgit Gaßner, Alexandra Gazinski, Jens Waschke, Albert Sickmann, Michaela Kuhn

**Affiliations:** 1 Institute of Physiology, University of WürzburgGermany; 2 Institute for Analytical SciencesDortmund, Germany; 3 Institute of Anatomy, University of WürzburgGermany; 4 Medizinisches Proteom-Center, Ruhr-University BochumGermany

**Keywords:** atrial natriuretic peptide, cyclic GMP, guanylyl cyclase A, mass spectrometry, phosphorylation

## Abstract

Atrial natriuretic peptide (ANP), via its guanylyl cyclase A (GC-A) receptor and intracellular guanosine 3′,5′-cyclic monophosphate production, is critically involved in the regulation of blood pressure. In patients with chronic heart failure, the plasma levels of ANP are increased, but the cardiovascular actions are severely blunted, indicating a receptor or postreceptor defect. Studies on metabolically labelled GC-A-overexpressing cells have indicated that GC-A is extensively phosphorylated, and that ANP-induced homologous desensitization of GC-A correlates with receptor dephosphorylation, a mechanism which might contribute to a loss of function *in vivo*. In this study, tandem MS analysis of the GC-A receptor, expressed in the human embryonic kidney cell line HEK293, revealed unambiguously that the intracellular domain of the receptor is phosphorylated at multiple residues: Ser487, Ser497, Thr500, Ser502, Ser506, Ser510 and Thr513. MS quantification based on multiple reaction monitoring demonstrated that ANP-provoked desensitization was accompanied by a complex pattern of receptor phosphorylation and dephosphorylation. The population of completely phosphorylated GC-A was diminished. However, intriguingly, the phosphorylation of GC-A at Ser487 was selectively enhanced after exposure to ANP. The functional relevance of this observation was analysed by site-directed mutagenesis. The substitution of Ser487 by glutamate (which mimics phosphorylation) blunted the activation of the GC-A receptor by ANP, but prevented further desensitization. Our data corroborate previous studies suggesting that the responsiveness of GC-A to ANP is regulated by phosphorylation. However, in addition to the dephosphorylation of the previously postulated sites (Ser497, Thr500, Ser502, Ser506, Ser510), homologous desensitization seems to involve the phosphorylation of GC-A at Ser487, a newly identified site of phosphorylation. The identification and further characterization of the specific mechanisms involved in the downregulation of GC-A responsiveness to ANP may have important pathophysiological implications.

**Structured digital abstract**

•MINT-7713870, MINT-7713887: *PMCA* (uniprotkb:P20020) and *GC-A* (uniprotkb:P18910) *colocalize* (MI:0403) by *fluorescence microscopy* (MI:0416)

## Introduction

Guanylyl cyclase A (GC-A, also known as natriuretic peptide receptor A) is a transmembrane receptor which synthesizes the intracellular second messenger guanosine 3′, 5′-cyclic monophosphate (cGMP) on binding of the ligands atrial natriuretic peptide (ANP) and B-type natriuretic peptide (BNP) to its extracellular domain. The NP/GC-A/cGMP system has important endocrine functions in the maintenance of arterial blood pressure and volume homeostasis [1]. Mice with global deletion of the genes encoding the GC-A receptor or ANP show marked hypervolemic hypertension and cardiac hypertrophy [2–5]. The relevance of these experimental observations to normal human physiology has been elegantly established by a recent genetic study which examined the association of common variants at the *ANP* and *BNP* gene loci with circulating concentrations of ANP/BNP and blood pressure [6]. The results demonstrated that genetically determined small variations in NP concentrations are associated with significant changes in blood pressure [6].

The GC-A receptor consists of an extracellular ligand-binding domain of approximately 441 amino acids (aa), a short membrane-spanning region (21 aa) and an intracellular portion (567 aa), containing a kinase homology (KH) domain, the dimerization domain and the C-terminal catalytic GC domain [1,7]. In the absence of ligand, GC-A forms homodimers or homotetramers, the KH domain is highly phosphorylated and the catalytic activity is tightly repressed [8–10]. On ANP binding, there is no change in the oligomeric state, but apparently a conformational change occurs which activates the cyclase domain [11]. Two cyclase domains form an active site and the second messenger cGMP is produced [11,12]. cGMP activates different intracellular signalling cascades which ultimately mediate the above-mentioned cardiovascular functions of ANP and BNP. The role of the KH domain is largely unknown. It presents approximately 30% homology to tyrosine kinases and approximately 20% homology to protein kinase A [13], but kinase activity has never been demonstrated. In the peptide-unliganded state, it inhibits the GC domain, a conclusion drawn from the observation that the KH domain deletion mutant is constitutively active [14]. Binding of a single peptide ligand between the two extracellular domains results in their relative reorientation, possibly relieving the inhibitory effect of the KH domains [11,15,16]. However, the mechanism by which KH domains mediate communication between the ligand-binding and GC domains is unclear.

In all patients with hypertensive cardiac hypertrophy and heart failure, the plasma levels of ANP and BNP are markedly increased, but the GC-A receptor-mediated functions are clearly diminished, indicating a receptor or postreceptor defect [1]. In view of the critical role of the NP/GC-A system in the moderation of blood pressure and volume [1–6], the identification of the specific mechanisms involved in the downregulation of GC-A activity may have important pathophysiological and clinical implications. Chronic exposure of the receptor to high concentrations of ANP can lead to homologous desensitization, which has been shown in many *in vitro* studies [17–20]. This desensitization procedure is probably a result of post-translational modifications, particularly dephosphorylation of the receptor [20]. Hence, on the basis of metabolic labelling experiments with GC-A-overexpressing HEK293 cells (human embryonic kidney cell line), Potter and Hunter [21,22] suggested the presence of six phosphorylated amino acids within a stretch of 15 membrane-near residues of the KH domain: Ser497, Thr500, Ser502, Ser506, Ser510 and Thr513. Mutations of these residues to Ala, mimicking the dephosphorylated version of the receptor, led to a diminished cGMP response of GC-A to ANP. In contrast, the conversion of these residues to glutamate, which mimics the negative charge of the phosphate moiety, restored receptor activity and ANP responsiveness [22]. From these experiments, Potter and Hunter [21,22] concluded that the phosphorylation of the KH domain is absolutely required for activation by ANP. In turn, dephosphorylation results in a desensitized receptor with diminished responsiveness to further hormonal stimulation. Thus, in contrast with G-protein-coupled receptors, which are desensitized by phosphorylation, phosphorylation seems to sensitize the GC-A receptor to ANP. However, the protein kinases and phosphatases responsible for this regulation have not been identified.

In this study, we aimed to verify unambig-uously the postulated phosphorylated residues and to characterize the so far unknown phosphorylated sites within the GC-A receptor. We enriched and purified FLAG-tagged GC-A from stably expressing HEK293 cells, as well as native GC-A from cultured murine cardiac microvascular endothelial cells, and analysed the phosphorylated residues by MS. The results confirm the phosphorylation of GC-A at the six amino acids previously suggested by Potter and Hunter [21,22], and reveal an additional neighbouring site of phosphorylation at Ser487. MS quantification based on multiple reaction monitoring was then applied to analyse the phosphorylation pattern of GC-A in HEK293 cells under basal conditions and after ANP-provoked homologous desensitization. Intriguingly, these results suggest that, in addition to the dephosphorylation of the previously postulated sites (Ser497, Thr500, Ser502, Ser506, Ser510 and Thr513), homologous desensitization of GC-A involves the phosphorylation of Ser487. Indeed, the results of site-directed mutagenesis, together with guanylyl cyclase receptor activity assays, support a role for the phosphorylation of this residue in the inhibitory regulation of GC-A activity.

## Results and Discussion

### The FLAG epitope does not modify the activity and subcellular localization of the GC-A receptor

First, it was necessary to prove that the N-terminal FLAG tag used to enrich GC-A from expressing HEK293 cells does not influence the activity of the receptor, its responsiveness to ANP or the subcellular localization.

**Fig. 1:**
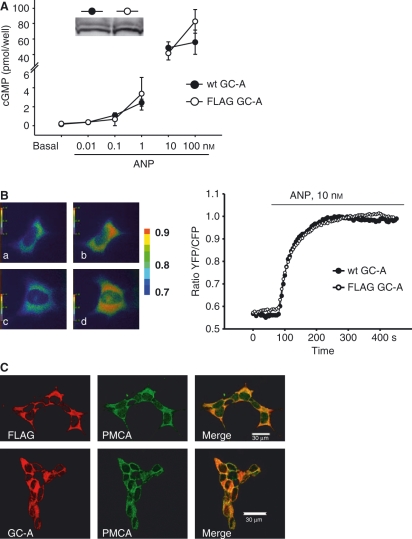
The N-terminal FLAG epitope does not alter the activity and subcellular localization of the GC-A receptor. (A) HEK293 cells expressing either the wild-type (wt) GC-A or FLAG-tagged GC-A receptor were incubated with ANP (10 pm to 100 nm, 10 min). Intracellular cGMP contents were quantified by RIA. Inset in (A): western blot analysis demonstrated similar expression levels of wt and FLAG-tagged GC-A. (B) FRET was used to monitor the kinetics and extent of cGMP formation in single HEK293 cells cotransfected with either wt GC-A or FLAG-tagged GC-A and cGMP indicator (pGES-DE2 [24]). Left: FRET images of two cells prior to and during incubation with ANP: wt GC-A with vehicle (a) and 10 nm ANP (b); FLAG-tagged GC-A with vehicle (c) and ANP (d). Right: representative ratiometric recordings of single-cell FRET signals. (C) Confocal immunofluorescence images of HEK293 cells transfected with wt or FLAG-tagged GC-A demonstrate the colocalization with PMCA.

The cGMP responses of HEK293 cells transiently transfected with either wild-type GC-A or FLAG-tagged GC-A receptor were quantified by RIA. ANP (10 pm–100 nm) evoked concentration-dependent increases in intracellular cGMP content, and these responses were similar in wild-type GC-A- and FLAG-tagged GC-A-expressing HEK293 cells (Fig. 1A). To monitor the kinetics and duration of cGMP formation by the two receptors in single intact cells, fluorescence resonance energy transfer (FRET) was used (Fig. 1B). The FRET biosensor pGES-DE2 responds to cGMP binding with a robust increase in FRET [23,24]. HEK293 cells coexpressing either wild-type GC-A or FLAG-tagged GC-A and the biosensor pGES-DE2 reacted to ANP with a strong increase in the FRET signal, indicating increases in [cGMP]_i_. The kinetics, duration and extent of cGMP formation in response to ANP were similar in wild-type GC-A- and FLAG-tagged GC-A-expressing HEK293 cells (Fig. 1B).

The subcellular localization of wild-type and FLAG-tagged GC-A in HEK293 cells was analysed by immunocytochemistry and confocal imaging. Figure 1C illustrates that both proteins colocalize with the plasma membrane-bound Ca^2+^-ATPase (PMCA).

Taken together, these data demonstrate that the FLAG epitope does not interfere with the cGMP responses of GC-A to ANP or the membrane localization, and therefore represents a good tool to facilitate the affinity purification of the receptor.

### Cell fractionation and immunoprecipitation lead to enrichment and purification of FLAG-tagged GC-A

**Fig. 2:**
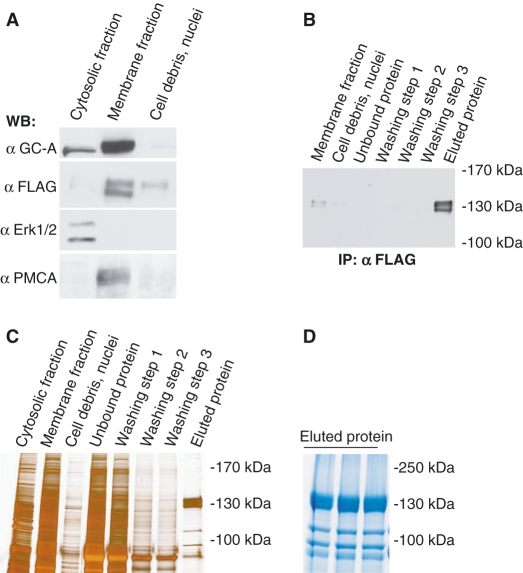
Enrichment and purification of the FLAG-tagged GC-A receptor from stably expressing HEK293 cells. (A) Cell fractionation and western blot analyses demonstrated that FLAG-tagged GC-A is predominantly localized in the plasma membrane of HEK293 cells. ERK1/2 and PMCA were used as markers for the cytosolic and membrane fractions, respectively. GC-A was detected with anti-GC-A serum and anti-FLAG IgG. (B) Western blot analysis demonstrated that immunoprecipitation of FLAG-tagged GC-A from the cell membrane fraction led to a 13-fold enrichment of the protein (2 μg protein per lane). (C) The silver-stained gel illustrates the step-wise purification of the receptor (10 μg protein per lane). (D) The immunoprecipitated GC-A protein was separated by SDS/PAGE. After Coomassie staining, the protein band at 130 kDa was excised and subjected to in-gel digestion with trypsin.

To enrich and purify the GC-A receptor for MS analyses, we used HEK293 cells stably expressing the FLAG-tagged GC-A receptor. The cells were fractionated and the membrane fraction was used to enrich the receptor by immunoprecipitation with anti-FLAG IgG. Western blot analyses with antibodies against PMCA, as a marker for the cell membrane, and extracellular signal-regulated kinase 1/2 (ERK1/2), as a marker for the cytosolic fraction, showed that cell fractionation led to a good separation of the membrane from the cytosolic proteins and from cell debris and nuclei (Fig. 2A). Western blot analyses with antibodies against FLAG and against the C-terminus of GC-A showed that the receptor was mainly localized in the membrane fraction (Fig. 2A). A small amount of GC-A was detected in the cytosolic fraction, which could be a result of incorrectly folded protein caused by cellular overexpression.

For each purification, the cell membrane fractions from 15 × 10 cm dishes (∼ 10^7^ cells per dish) were combined and subjected to immunoprecipitation with anti-FLAG IgG coupled to agarose beads. Bound proteins were eluted by the addition of synthetic triple-FLAG peptide. Western blot analyses (Fig. 2B) and silver-stained gels (Fig. 2C) demonstrated that this procedure resulted in a marked enrichment (by approximately 13-fold; Fig. 2B) and purification (Fig. 2C) of GC-A. The eluted GC-A protein was precipitated by trichloroacetic acid, separated by SDS/PAGE and visualized by colloidal Coomassie staining (Fig. 2D). The protein band corresponding to GC-A (MW ∼ 130 kDa) was excised and digested with trypsin.

### MS analyses of the GC-A receptor, exogenously expressed in HEK293 cells, reveal seven phosphorylated residues within the KH domain

On the basis of experiments with GC-A-overexpressing HEK293 cells, metabolic labelling with [^32^P]orthophosphate and site-directed mutagenesis, Potter and Hunter [21,22] described six phosphorylated residues within the KH domain. Here, we applied MS to verify unambiguously these postulated sites and to search for additional phosphorylation sites within the GC-A receptor without metabolic labelling and therefore without stressing the cells [25,26]. Trypsin digestion and liquid chromatography (LC)-MS/MS yielded 61% sequence coverage of the 130 kDa rat GC-A, with highest sequence coverage for the N-terminus (positions 22–200 of the extracellular domain), the KH domain and the last part of the catalytic domain of GC-A (Fig. S1). However, in these analyses, we did not detect phosphopeptides, indicating that they are less abundant than nonphosphorylated tryptic GC-A peptides.

**Fig. 3:**
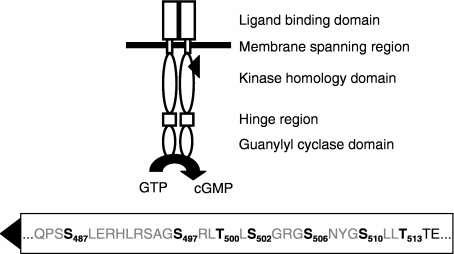
Scheme illustrating the domains of GC-A and the positions of the phosphorylated amino acids (in bold). The numbers within the sequence depict the positions of these amino acids within mature rat GC-A [7].

**Fig. 4:**
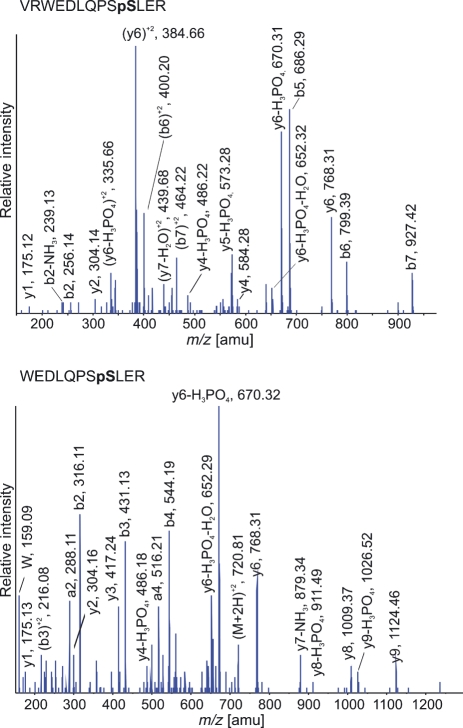
Fragment ion spectra of the phosphopeptides VRWEDLQPSpSLER and WEDLQPSpSLER, both representing the phosphorylated Ser487 of the GC-A receptor.

To enhance the sensitivity of our measurements, TiO_2_ affinity chromatography was used to enrich the putative phosphopeptides. Detection was performed by nano-LC-MS/MS. The combined results of two independent biological experiments (two purifications of GC-A, four enrichments by TiO_2_) are summarized in Table 1, including only phosphopeptides which were reproducibly detected and manually validated. Further potential phosphopeptides which did not pass this manual validation are not included. The spectra are shown in Fig. S2. As depicted in Table 1, all detected tryptic phosphopeptides were derived from the KH domain of GC-A, and all six phosphorylation sites previously deduced by Potter and Hunter [21,22] from experiments with metabolically labelled HEK293 cells were verified: Ser497, Thr500, Ser502, Ser506, Ser510, Thr513 (Fig. 3). In addition, one novel site of phosphorylation was identified at Ser487 within the KH domain (Table 1; Fig. 3). The mass spectra of the two tryptic peptides (478–490 and 480–490 of GC-A) containing this new phosphorylation site are shown in Fig. 4.

**Table 1:** Rat GC-A (Sprot accession number P18910) tryptic phosphopeptides detected by MS after purification of FLAG-tagged GC-A from overexpressing HEK293 cells. The phosphorylation sites are marked in bold and listed separately. The numbers refer to the respective positions within the mature GC-A protein [7]. The abbrevations used are as follows: *z*, precursor charge; *m*/*z*, mass-to-charge ratio; *M*_r_(exp), experimental mass; *M*_r_(calc), theoretical mass; Delta, mass deviance *M*_r_(exp) – *M*_r_(calc); ND, not detected.

Position	Peptide sequence	Phosphorylation site(s)	Score	*m*/*z*	*z*	*M*_r_(exp)	*M*_r_(calc)	Delta	Mass analyser
478–490	R.VRWEDLQPS**pS**LER.H	S487	37	565.66	3	1693.96	1693.78	0.18	QstarElite
480–490	R.WEDLQPS**pS**LER.H	S487	39	720.40	2	1438.72	1438.61	0.11	QstarElite
494–504	R.SAG**pS**RLTLSGR.G	Ser497	33	395.54	3	1183.60	1183.57	0.03	Qtrap
494–504	R.SAGSRL**pT**LSGR.G	ND							
494–504	R.SAGSRLTL**pS**GR.G	ND							
494–504	R.SAG**pS**RL**pT**LSGR.G	Ser497 and Thr500	21	422.19	3	1263.54	1263.55	−0.01	Qtrap
494–504	R.SAG**pS**RLTL**pS**GR.G	Ser497 and Ser502	34	632.83	2	1263.64	1263.54	0.10	QstarElite
494–504	R.SAGSRL**pT**L**pS**GR.G	Thr500 and Ser502	38	422.21	3	1263.61	1263.54	0.07	Qtrap
494–504	R.SAG**pS**RL**pT**L**pS**GR.G	Ser497, Thr500 and Ser502	32	672.71	2	1343.41	1343.50	−0.09	Qtrap
499–504	R.L**pT**LSGR.G	Thr500	34	363.68	2	725.34	725.35	−0.01	Qtrap
499–504	R.LTL**pS**GR.G	Ser502	28	363.68	2	725.33	725.35	−0.02	Qtrap
499–504	R.L**pT**L**pS**GR.G	Thr500 and Ser502	26	403.69	2	805.37	805.31	0.06	QstarElite
505–523	R.G**pS**NYGSLLTTEGQFQVFAK.T	Ser506	84	1063.89	2	2125.77	2125.97	−0.20	Qtrap
505–523	R.GSNYG**pS**LLTTEGQFQVFAK.T	Ser510	100	1063.91	2	2125.81	2125.97	−0.16	Qtrap
505–523	R.GSNYGSLL**pT**TEGQFQVFAK.T	Thr513	57	709.74	3	2126.20	2125.97	0.23	QstarElite
505–523	R.G**pS**NYG**pS**LLTTEGQFQVFAK.T	Ser506 and Ser510	78	1103.92	2	2205.83	2205.94	−0.11	Qtrap
505–523	R.G**pS**NYGSLL**pT**TEGQFQVFAK.T	ND							
505–523	R.GSNYG**pS**LL**pT**TEGQFQVFAK.T	Ser510 and Thr513	87	736.29	3	2205.84	2205.94	−0.10	Qtrap
505–523	R.G**pS**NYG**pS**LL**pT**TEGQFQVFAK.T	ND							

As depicted in Table 1, in these experiments, both fully and partially phosphorylated tryptic peptides were detected. For instance, the tryptic peptide SAGSRLTLSGR (residues 494–504) can be phosphorylated at three sites (Ser497, Thr500 and Ser502). Only one of the three possible single phosphorylated peptides (phosphorylated at Ser497) was detected (Table 1). When this peptide is phosphorylated at Ser497, upstream tryptic cleavage is considerably reduced at Arg498 (SAGSR | LTLSGR) because of steric hindrance by the phosphate. This might explain why the phosphorylated peptide SAG**pS**R was not detected, even by multiple reaction monitoring. When not phosphorylated at Ser497, the peptide SAGSRLTLSGR can be cleaved by trypsin, and therefore the other two phosphorylation sites (Thr500 and Ser502) were detected in the peptides L**pT**LSGR and LTL**pS**GR. The triply phosphorylated peptide SAGpSRLpTLpSGR was also detected. When this peptide is phosphorylated at two or all three possible positions, tryptic cleavage is also reduced. Taken together, these results clearly demonstrate the phosphorylation of GC-A at Ser497, Thr500 and Ser502 (Table 1).

The tryptic peptide GSNYGSLLTTEGQFQVFAK (residues 505–523) containing the three additional phosphorylation sites suggested by Potter and Hunter [21,22] (Ser506, Ser510 and Thr513) was detected as a singly and dually phosphorylated form (Table 1); however, the fully phosphorylated peptide was not detected. This may be a result of a combination of reduced suitability of TiO_2_ enrichment for multiply phosphorylated peptides and the generally lower ionization/detection properties of highly phosphorylated peptides in ESI-MS/MS. Here, perhaps the use of alternative enrichment strategies, such as immobilized metal ion affinity chromatography, in conjunction with electron transfer dissociation for fragmentation, might enable the detection.

Lastly, in addition to the tryptic phosphopeptides containing the previously postulated sites [21,22], we detected two additional phosphopeptides (residues 478–490 and 480–490), revealing a so far unknown phosphorylation at the neighbouring Ser487 (Table 1). The corresponding mass spectra are illustrated in Figs 4 and S2.

### MS analyses of the GC-A receptor, endogenously expressed in endothelial cells, confirm the newly identified site of phosphorylation at Ser487

Next, we analysed the phosphorylation pattern of the native GC-A receptor endogenously expressed in cultured murine microvascular myocardial endothelial cells [27]. We used these cells because, compared with other cell culture systems, they have comparatively high endogenous GC-A expression levels, and because ANP modulates important cellular functions, such as permeability and angiogenic growth (J. Schröter *et al.*, unpublished observations). Western blot analyses showed that their GC-A expression levels were approximately 100-fold lower than those of stably transfected HEK293 cells (not shown). Native GC-A was enriched from plasma membrane fractions with an antibody directed against the C-term of the receptor. Similar to the experiments with HEK293 cells, we detected various overlapping tryptic GC-A phosphopeptides derived from the N-terminus of the KH domain. The tandem mass spectra, which are illustrated in Fig. S3, revealed the following phosphorylated residues: Ser 487, Ser497 and Thr500. Because of the low abundance of tryptic GC-A phosphopeptides, we could not confirm the additional four phosphorylation sites which were identified in the experiments with HEK293 cells (Ser502, Ser506, Ser510, Thr513). However, these analyses demonstrate, for the first time, the phosphorylation of the endogenous (untransfected) native GC-A receptor. Most importantly, they confirm the newly identified site of phosphorylation at Ser487.

### Multiple reaction monitoring reveals that homologous desensitization of the GC-A receptor in HEK293 cells is associated with a complex phosphorylation pattern

The second part of our study aimed to analyse, in HEK293 cells, the changes in the phosphorylation pattern of GC-A accompanying its homologous desensitization. Within each experiment, GC-A-expressing HEK293 cells were incubated with ANP (100 nm, 1 h) or remained untreated (15 dishes per condition; three independent experiments). For semiquantitative analyses of tryptic GC-A phosphopeptides enriched from ANP-treated relative to untreated cells, the technique of multiple reaction monitoring was applied. This allows a label-free quantification of phosphorylated tryptic GC-A peptides by peak area comparison [28,29]. After immunoprecipitation and trypsin digestion, the samples were spiked with two synthetic phosphopeptides before TiO_2_ enrichment, and with two additional synthetic peptides before MS. As shown in Table 2, within each experiment, the recovery of these standard peptides from untreated and ANP-treated samples was nearly identical, ensuring comparability.

**Table 2:** Relative quantification of the phosphorylated tryptic peptides by multiple reaction monitoring. The ratio of multiple reaction monitoring peak areas after ANP pretreatment versus control conditions was calculated. Shown are the mean values ± SEM of the ratios obtained in three independent biological experiments (in total eight enrichments, eight analyses with multiple reaction monitoring). The fully phosphorylated tryptic peptides are marked by an asterisk. Standard synthetic (phospho)peptides were added to the samples as described.

		Ratios of signal intensities of phosphopeptides obtained from ANP-pretreated versus untreated HEK293 cells	
Phosphopeptides	Phosphorylation site(s)	Mean	SEM
WEDLQPS**pS**LER	Ser487	9.55	1.31
VRWEDLQPS**pS**LER	Ser487	9.72	1.58
LTL**pS**GR	Ser502	2.30	0.37
L**pT**LSGR	Thr500	1.79	0.23
L**pT**L**pS**GR*	Thr500 and Ser502	0.50	0.09
SAG**pS**RLTLSGR	Ser497	0.94	0.31
SAGSRL**pT**L**pS**GR	Thr500 and Ser502	3.64	0.99
SAG**pS**RLTL**pS**GR	Ser497 and Ser502	2.60	0.48
SAG**pS**RL**pT**LSGR	Ser497 and Thr500	2.63	0.44
SAG**pS**RL**pT**L**pS**GR*	Ser497, Thr500 and Ser502	0.19	0.07
GSNYG**pS**LLTTEGQFQVFAK	Ser510	1.61	0.36
G**pS**NYGSLLTTEGQFQVFAK	Ser506	2.51	0.68
GSNYGSLL**pT**TEGQFQVFAK	Thr513	1.72	0.39
G**pS**NYG**pS**LLTTEGQFQVFAK	Ser506 and Ser510	0.94	0.29
GSNYG**pS**LL**pT**TEGQFQVFAK	Ser510 and Thr513	1.25	0.76
Synthetic standard peptides			
VGGHAAEYGAEALER		0.93	0.09
TEREDLIAYLK		0.94	0.10
VKVDEVGGEALGR		1.07	0.05
EFTPVLQADFQK		0.98	0.04
Synthetic standard phosphopeptides			
DIG**pS**E**pS**TEDQAEDIK	Ser4 and Ser6	1.22	0.34
NSLVTQDD**pT**FKDK	Thr9	1.07	0.07

**Fig. 5:**
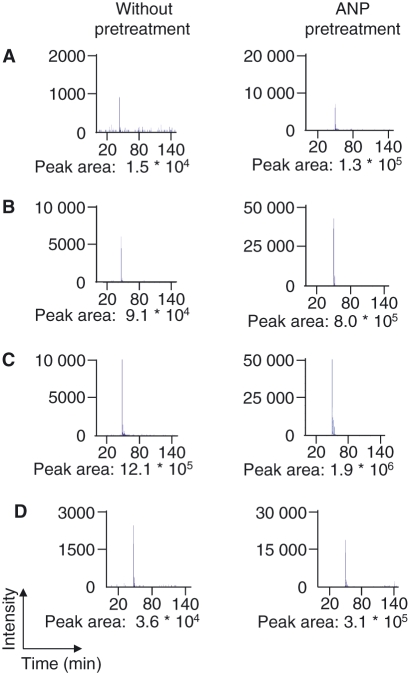
Multiple reaction monitoring was used for semiquantitative analysis of the tryptic GC-A phosphopeptides obtained from ANP- relative to vehicle-treated (control) FLAG-tagged GC-A-expressing HEK293 cells. Four transitions obtained from MS/MS spectra of the phosphopeptide WEDLQPSpSLER were chosen to analyse the peptide content in ANP-treated relative to untreated samples. Peak areas, which are depicted under the spectra, showed an approximately nine-fold increase in ANP-treated versus untreated samples, indicating an increase in peptide amount (sum of peak areas without pretreatment, 352 000; sum of peak areas after ANP pretreatment, 3 140 000). (A) Transition 720.3/486.3. (B) Transition 720.3/670.4. (C) Transition 720.3/768.4. (D) Transition 720.3/288.1.

**Fig. 6:**
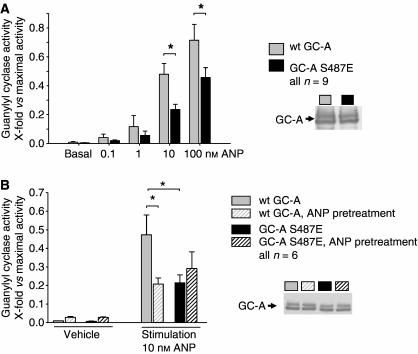
The impact of the phosphorylation of the GC-A receptor at Ser487 on the responsiveness and homologous desensitization of the receptor was characterized by site-directed mutagenesis followed by guanylyl cyclase activity assays. Crude membranes prepared from HEK293 cells expressing wild-type (wt) GC-A or GC-A S487E were incubated with vehicle, ANP or detergent (Triton X-100). cGMP production was measured by RIA [fmol cGMP·(μg protein)^−1^·min^−1^]. All values were calculated as *X*-fold of the maximal (Triton X-100-induced) activity (means ± SEM). The western blots shown in the insets demonstrate similar expression levels of wt and mutated GC-A (all 10 μg protein per lane). (A) ANP evoked concentration-dependent increases in GC-A activity. The GC-A S487E mutant showed significantly reduced responsiveness to ANP (*n* = 9 from three independent experiments). (B) HEK293 cells were pretreated with ANP (100 nm, 1 h) or vehicle before the preparation of cell membranes. ANP pretreatment decreased significantly the cGMP response of wt GC-A to subsequent stimulation with 10 nm ANP, indicating homologous desensitization. The GC-A S487E mutant showed a significantly diminished cGMP response to 10 nm ANP, which was not further inhibited by ANP pretreatment (*n* = 6 from three independent experiments).

Table 2 summarizes the results of three independent biological experiments (mean values ± SEM of eight independent enrichments, eight analyses by multiple reaction monitoring). The results are presented as the ratio of multiple reaction monitoring peak areas for tryptic GC-A phosphopeptides detected after purification of the FLAG-tagged GC-A receptor from HEK293 cells either pretreated with ANP (100 nm, 1 h) or untreated (controls). In each experiment, both conditions (±ANP) were compared. Western blot results demonstrated that the amount of GC-A in cells did not change significantly after 1 h of ANP stimulation (Fig. 6B). However, as described in the next section, ANP pretreatment markedly reduced the responsiveness of GC-A to subsequent ANP stimulation, indicating homologous desensitization of the receptor. Figure 5 illustrates four examples of multiple reaction monitoring scans showing considerable changes in peptide abundance on ANP treatment. For instance, the sum of the peak areas of the four transitions of the peptide WEDLQPS**pS**LER, which contains the newly discovered phosphorylation site Ser487, on average was increased by approximately nine-fold in ANP-treated samples, indicating an increased amount of peptide (Fig. 5). Taken together, the results from multiple reaction monitoring revealed that the amount of partially phosphorylated tryptic peptides containing the residues Ser497, Thr500 and Ser502 was increased by approximately two-fold after ANP pretreatment (Table 2). In contrast, the amount of completely phosphorylated peptides was decreased by 50% or more (Table 2, asterisks). These observations indicate that homologous desensitization of GC-A was concomitant with a reduction in the population of GC-A receptors fully phosphorylated at positions Ser497, Thr500 and Ser502. This is in line with the observations of Potter *et al.* [21,22,30] in metabolically labelled HEK293 cells, which showed a marked dephosphorylation of GC-A after ANP pretreatment. Our present results extend these observations, suggesting that the cGMP responses of GC-A to ANP are already blunted when the receptor population is not totally, but only partly, dephosphorylated.

Unfortunately, the results obtained for the tryptic phosphopeptide spanning positions 505–523 of GC-A, which contains the phosphorylation sites Ser506, Ser510 and Thr513, are difficult to interpret, because, as mentioned above, the fully phosphorylated version was never detected. Nevertheless, Table 2 demonstrates that the relative amount of partially phosphorylated peptides was increased in samples obtained from ANP-treated HEK293 cells (when compared with untreated cells), again suggesting that the population of receptors fully phosphorylated at these three additional positions was diminished.

Most remarkably, as already mentioned above, the amount of the two tryptic GC-A phosphopeptides containing the newly detected phosphorylation at Ser487 was strongly increased after ANP pretreatment, by nearly nine-fold (Table 2, top). This suggests that this phosphorylation site, in contrast with the others, is phosphorylated during homologous desensitization. Hence, homologous desensitization of GC-A is associated with a complex pattern of (de)phosphorylation of the receptor, with a selective prominent increase in the phosphorylation of GC-A at Ser487.

### Site-directed mutagenesis indicates that phosphorylation of the GC-A receptor at Ser487 inhibits its activity

To analyse whether the phosphorylation of Ser487 modulates the responsiveness of GC-A to ANP, this residue was substituted with glutamate (S487E) to mimic constitutive phosphorylation by substituting Ser487 with an amino acid containing a negatively charged side-chain. The effect of this substitution on GC-A activity was evaluated in guanylyl cyclase assays performed with crude membranes from GC-A-expressing HEK293 cells. The membranes were incubated with ANP, and cGMP formation was measured by RIA. We confirmed, by immunoblotting, that mutant and wild-type GC-A receptors were expressed in similar amounts (see insets in Fig. 6A,B). In addition, to account for small differences in the expression level of the two variants, we normalized the basal and ANP-stimulated activity data with the respective maximal, Triton-stimulated, GC-A activity [12,17,21,22].

Wild-type GC-A responded to ANP with concentration-dependent increases in cGMP production (Fig. 6A). In comparison, the cGMP responses of the mutant GC-A S487E to ANP were markedly blunted (Fig. 6A). One possible explanation for this reduced responsiveness is the different steric conformation of glutamate and phosphate, which might impose different constraints on protein conformation. However, it should be noted that, in previous studies, mutations of the other phosphorylated residues (Ser497, Thr500, Ser502, Ser506, Ser510 and Thr513) to glutamate did not reduce, but enhanced, receptor activity and responsiveness, indicating that glutamate adequately mimicked the phosphorylated state [21,22]. Taken together, our observations showing that the cGMP responses of GC-A S487E to ANP were markedly impaired, and that ANP-induced desensitization of GC-A was accompanied by greatly increased phosphorylation at Ser487, support a role for the phosphorylation of this residue in the inhibitory regulation of GC-A activity.

To follow this hypothesis, we tested the influence of the substitution of Ser487 with glutamate on the process of homologous desensitization of GC-A. HEK293 cells expressing wild-type GC-A or GC-A S487E were incubated with ANP (100 nm, 1 h) or remained untreated. After vigorous washing, cell membranes were prepared for the assay of guanylyl cyclase activity. As shown in Fig. 6B, ANP pretreatment markedly diminished the cGMP responses of wild-type GC-A to a subsequent stimulation with 10 nm ANP. Notably, western blotting demonstrated that ANP pretreatment did not affect the expression levels of GC-A (see inset in Fig. 6B). Hence, in agreement with previous studies [22,30], this experimental condition led to a pronounced homologous desensitization of the GC-A receptor, which was not caused by receptor internalization or degradation. Once more, the experiments with HEK293 cells expressing GC-A S487E led to a completely different result. As mentioned above, already under basal conditions (without ANP pretreatment), the responsiveness of this mutated receptor to ANP was markedly blunted. In fact, the cGMP responses of GC-A S487E to 10 nm ANP were similar to the responses of the desensitized wild-type GC-A receptor (Fig. 6B). In addition, GC-A S487E was not further inactivated by ANP pretreatment (Fig 6B). These observations corroborate our hypothesis that the phosphorylation at Ser487 could be involved in the desensitization of the receptor. We propose that ANP-induced phosphorylation of Ser487 either directly induces a conformational change which inhibits GC-A activity or creates a docking site for a phosphatase which catalyses the dephosphorylation of the neighbouring residues.

In summary, our study demonstrates, for the first time, the phosphorylation pattern of the GC-A receptor in overexpressing HEK293 cells by MS. Seven phosphorylated amino acids within the KH domain were unambiguously detected: Ser497, Thr500, Ser502, Ser506, Ser510 and Thr513 (which were previously indicated by Potter *et al.* [21,22,30]), and a novel site of phosphorylation at the neighbouring proximal Ser487. Several of these phosphorylation sites (Ser487, Ser497 and Thr500) were verified in murine microvascular endothelial cells endogenously expressing the GC-A receptor. Remarkably, these studies confirmed the newly identified site of phosphorylation of native, endogenous GC-A at Ser487. In HEK293 cells, homologous desensitization of GC-A was accompanied by a diminished population of completely phosphorylated GC-A receptors, but a selective and dramatic increase in the phosphorylation at Ser487. Lastly, a functional role for phosphorylation at Ser487 in the ANP-induced inactivation of GC-A has been demonstrated by engineering a mutation that mimics the phosphorylated form of this residue. Application of the kinase–substrate interaction prediction algorithms NetphosK and NetworKIN validated our MS results, revealing a high probability of phosphorylation of GC-A at Ser487. These computational analyses indicated that this site conforms to the consensus motifs for the DNA-dependent protein kinase catalytic subunit and for cyclin-dependent kinase 2. The DNA-dependent protein kinase catalytic subunit plays an important role in the repair of DNA double-strand breaks. The mitotic cyclin-dependent kinase 2 is involved in the regulation of progression through the cell cycle. Nothing is known about the role of these kinases in the control of arterial blood pressure, and therefore both are unlikely to participate in the regulation of the responsiveness of the GC-A receptor to ANP. Hence, although the critical role of phosphorylation in the regulation of GC-A enzymatic activity and responsiveness to ANP has been clearly demonstrated by the present and published studies [21,22,30], the protein kinases and phosphatases that add phosphate to and remove it from the receptor have yet to be identified. To follow this important question, in future studies, we will attempt to generate phosphospecific antibodies to further characterize the modulation and function of pGC-A Ser487 and the (patho)physiological relevance *in vivo*.

One important limitation to our study was the low abundance of phosphorylated tryptic peptides in contrast with unphosphorylated peptides, which made it difficult to detect the phosphorylation sites. This limitation was partly solved by using TiO_2_ affinity chromatography. In addition, although the whole receptor was scanned by MS, the results obtained with GC-A purified from overexpressing HEK293 cells only provided 61% coverage of the protein sequence (Fig. S1). As a result of this limitation, we cannot rule out the existence of additional phosphorylation sites within the GC-A receptor, which, for instance, might not be suited to tryptic digestion, or which might reveal poor fragmentation on collision-induced dissociation. In view of the important cardiovascular actions of the ANP/GC-A system, the identification and further characterization of the post-translational modifications and of the regulatory proteins involved in the downregulation of GC-A activity may have important pathophysiological implications. We hope that the methods and observations described in this article will be helpful in facilitating some of these discoveries.

## Experimental procedures

### Determination of intracellular cGMP by RIA and FRET

HEK293 cells were maintained in Dulbecco’s modified Eagle’s medium supplemented with 10% fetal bovine serum. The cells were transiently transfected with the plasmids pCMV5-GC-A (encoding wild-type rat GC-A cDNA) or pCMV5-FLAG-GC-A (encoding N-terminally FLAG-tagged rat GC-A) using FuGene transfection reagent, according to the manufacturer’s recommendations (Roche Applied Science, Mannheim, Germany). One day later, the cells were serum starved for 16 h and then stimulated with ANP (rat ANP; Bachem, Heidelberg, Germany) for 10 min in the presence of the phosphodiesterase inhibitor 3-isobutyl-1-methylxanthine (0.5 mm; Sigma-Aldrich, Deisenhofen, Germany). Intracellular cGMP was measured by RIA [23]. In addition, FRET was used to monitor, in real time, the kinetics and extent of cGMP formation in intact HEK293 cells cotransfected with wild-type or FLAG-tagged GC-A and the cGMP indicator pGES-DE2 [23,24].

### Fluorescence microscopy

HEK293 cells, stably expressing wild-type GC-A or FLAG-tagged GC-A receptors, were grown in chamber slides, fixed in ice-cold 4% paraformaldehyde, permeabilized with 0.2% Triton X-100 and blocked with 5% fetal bovine serum in phosphate-buffered saline solution (NaCl/P_i_). After incubation with primary antibodies against GC-A (generated in our laboratory against the C-terminus of GC-A, CKGKVRTYWLLGERGSSTRG), FLAG (Sigma-Aldrich) or PMCA (clone 5F10, Sigma-Aldrich), the cells were incubated with fluorescence-conjugated secondary antibodies.

### Enrichment of FLAG-tagged GC-A receptor by cell fractionation and immunoprecipitation

HEK293 cells, stably expressing FLAG-tagged GC-A, were cultivated in Dulbecco’s modified Eagle’s medium supplemented with 10% fetal bovine serum and zeocin (300 μg·mL^−1^). For separation of the soluble and particulate cellular proteins, 15 dishes (10 cm) (∼ 15 × 10^7^ cells) were washed twice with NaCl/P_i_ and lysed on ice to separate the cytosol according to the manufacturer’s instructions (CF-Cyt, NanoTools, Teningen, Germany). After centrifugation at 1000 ***g*** for 5 min at 4 °C, the supernatant containing the cytosolic fraction was removed. The membrane pellet was lysed in Tris buffer of the following composition: 20 mm Tris/HCl, pH 7.4, 150 mm NaCl, 1 mm Na_2_EDTA, 1 mm EGTA, 1.5 mm Na_2_H_2_P_2_O_7_ and 1% Triton X-100, supplemented with protease and phosphatase inhibitor cocktails (Roche Applied Science). The latter contains sodium orthovanadate, sodium pyrophosphate, among others, and inhibits the classes of acid and alkaline phosphatases, as well as serine/threonine (PP1, PP2A, PP2B) and tyrosine protein phosphatases. Because it was shown in experiments with metabolically labelled HEK293 cells that this combination inhibits the dephosphorylation of GC-A [22,30], these phosphatase inhibitors were also included in all subsequent purifications. After incubation with lysis buffer for 30 min on ice with vigorous vortexing, the cell debris and nuclei were pelleted by centrifugation at 2000 ***g*** for 10 min at 4 °C. The resulting supernatant contained the solubilized cell membranes.

FLAG-tagged GC-A receptor was enriched from membrane fractions by incubation with anti-FLAG IgG coupled to agarose beads (M2 agarose, Sigma-Aldrich) for 2 h at 4 °C, followed by extensive washing with Tris buffer. The protein was eluted in Tris buffer containing 600 μg·mL^−1^ synthetic triple FLAG peptide (Sigma-Aldrich). The protein concentration was determined by the bicinchoninic acid protein assay (Interchim, Mannheim, Germany).

### Western blot analyses and silver staining

Aliquots of the extracted and immunoprecipitated proteins were incubated with Laemmli buffer and separated by SDS/PAGE. For western blotting, antibodies were against PMCA, the mitogen-activated protein kinase ERK1/2 (Cell Signaling Technology, Frankfurt, Germany), FLAG or GC-A. Immunoreactive proteins were detected by chemiluminescence using ECL (Thermo Scientific, Schwerte, Germany). SDS/PAGE and silver staining were used to assess the purity of the fractions.

### Preparation of samples for TiO_2_ affinity chromatography

For MS, the immunoprecipitated FLAG-tagged GC-A receptor was purified by SDS/PAGE (8% gel), stained with Coomassie, and the GC-A band (apparent MW ∼ 130 kDa) was excised from the gel. In-gel digestion with trypsin (Promega, Mannheim, Germany) was conducted according to Wilm *et al.* [31]. After elution of the tryptic peptides, phosphorylated peptides were further enriched by TiO_2_ affinity chromatography. The peptide eluate was concentrated under vacuum and the nearly dried peptides were incubated in loading buffer (80% acetonitrile, 2.5% trifluoroacetic acid, saturated with phthalic acid) containing 200 μg of TiO_2_ beads per excised protein band for 1 h at room temperature. The beads were washed twice with loading buffer, washing buffer (80% acetonitrile, 0.1% trifluoroacetic acid) and 0.1% trifluoroacetic acid, and the phosphopeptides were eluted in three steps with 200, 300 and 400 mm NH_4_OH containing 30% acetonitrile. The eluted peptide solution was immediately acidified with formic acid to pH 4.0.

### Mass spectrometry

Nano-LC-MS/MS analyses were carried out using Qtrap™ 4000 and QstarElite mass analysers (Applied Biosystems, Darmstadt, Germany) coupled to Ultimate 3000 nano-HPLC systems (Dionex, Amsterdam, the Netherlands). The peptides were concentrated and separated as described previously [32]. Full MS scans from 350 to 2000 *m*/*z* were acquired, and the four (Qtrap™ 4000) or three (Qstar® XL) most intensive signals were subjected to MS/MS, taking into account a dynamic exclusion. Transformation of raw data into mfg format was performed as described previously [28]. Tandem mass spectra were searched against the Swiss-Prot database (June 2009, 497 293 entries) using Mascot 2.2.0.0. The following search parameters were used: taxonomy was set to *Rattus norvegicus* (7419 sequences); trypsin as protease with a maximum of two missed cleavage sites; carbamidomethylation of Cys (+57.02 Da) as fixed modification; oxidation of Met (+15.99 Da) and phosphorylation of Ser/Thr/Tyr (+79.96 Da) as variable modifications. For the QTrap, mass tolerances were set to 0.4 Da for MS and MS/MS, whereas, for the QstarElite, mass tolerances were set to 0.25 Da for MS and 0.5 Da for MS/MS. Only phosphopeptides with a probability of *P* < 0.05 for a random hit were considered and, furthermore, manually validated as described previously [28].

### Enrichment of the GC-A receptor from murine microvascular myocardial endothelial cells

Murine myocardial endothelial cells were maintained in Dulbecco’s modified Eagle’s medium with 10% fetal bovine serum [27]. For overnight starvation, the medium was reduced to 1% fetal bovine serum. Forty-five 10 cm dishes (45 × 10^7^ cells) were used for the separation of cytosolic, plasma membrane and nuclear fractions, as described above. Endogenously expressed GC-A was enriched by incubation of the combined plasma membrane fractions with affinity-purified anti-GC-A serum (generated in our laboratory) coupled to agarose beads (Sigma-Aldrich) for 4 h at 4 °C, followed by extensive washing with Tris buffer (see above). The protein was eluted by pH shift (to pH 2.5) and the acidic eluate was immediately neutralized to pH 7.4 using 0.5 m Tris, pH 9.0, with 1.5 m NaCl. Gel purification by electrophoresis on 8% SDS/PAGE, in-gel digest with trypsin, TiO_2_ affinity chromatography and nano-LC-MS/MS analyses were carried out as described above, except that taxonomy was set to *Mus musculus.*

### Multiple reaction monitoring

To characterize the changes in the phosphorylation pattern of the GC-A receptor exogenously expressed in HEK293 cells after ANP-provoked homologous desensitization, the tryptic phosphopeptides were quantified by multiple reaction monitoring [29]. Appropriate transitions for multiple reaction monitoring were manually selected from representative fragment ion spectra of the respective phosphopeptides from previous MS/MS analyses. To improve the level of confidence, three to five fragments per parent ion were selected. Collision energies were assigned as described previously [33]. A total of 68 multiple reaction monitoring transitions were used and, for all monitored transitions, the dwell time was set to 20 ms, whereas the total cycle time was not allowed to exceed approximately 1 s for a single analysis [33].

For these analyses, HEK293 cells stably expressing FLAG-tagged GC-A receptor were serum starved for 16 h and were then incubated with ANP (0.1 μm, 1 h, 37 °C) or remained untreated (in each experiment, 15 × 10 cm dishes were used for each condition; three independent experiments). Immunoprecipitation of GC-A, in-gel digest with trypsin, TiO_2_ affinity chromatography and nano-LC-MS/MS analyses were carried out as described above. Data interpretation and quantification were accomplished using multiquant 1.0 software (Applied Biosystems) [33]. Estimates of the relative abundance of phosphorylated tryptic peptides of GC-A isolated from HEK293 cells treated with ANP relative to untreated cells were determined from the peak areas of the multiple reaction monitoring scans. Data are presented as the ratios of the multiple reaction monitoring peak areas of the phosphorylated tryptic GC-A peptides obtained from ANP-pretreated to untreated HEK293 cells. The samples were spiked with two synthetic standard phosphopeptides before TiO_2_ affinity chromatography and with two additional synthetic peptides shortly before starting the MS analyses (Table 2). The multiple reaction monitoring scans of these standard peptides were used to ensure that TiO_2_ enrichment and MS conditions were equal for tryptic GC-A phosphopeptides obtained from untreated and ANP-treated cells.

### Site-directed mutagenesis and guanylyl cyclase assay

Rat FLAG-tagged GC-A in pCMV5 vector served as template for the site-directed substitution of the novel phosphorylation site Ser487 with glutamate. The oligonuclotide primers and details of PCRs are depicted in Table S1. The mutation and absence of unwanted mutations were verified by sequencing. HEK293 cells were transiently transfected with the wild-type or GC-A S487E expression constructs using FuGene (Roche Applied Science). Transfected cells were serum starved for 16 h prior to ANP (100 nm, 1 h) or vehicle (NaCl/P_i_) exposure (48 h after transfection). To prepare crude membranes, the cells of one 10 cm dish (∼10^7^ cells) were washed twice with NaCl/P_i_, lysed in Hepes buffer [50 mm Hepes, pH 7.4, containing 100 mm NaCl, 10% glycerol, protease and phosphatase inhibitor cocktails (Roche Applied Science)], and the lysates were pelleted by centrifugation (16 000 ***g***, 10 min, 4 °C). The protein concentration was determined by bicinchoninic acid protein assay. All guanylyl cyclase activity assays were carried out at 37 °C in 50 mm Hepes buffer, pH 7.4, containing 50 mm NaCl, 5% glycerol, 0.05% BSA, 1 mm 3-isobutyl-1-methylxanthine, 2 mm GTP, 30 mm creatine phosphate and 1.5 units·mL^−1^ (0.3 units per assay) creatine phosphokinase [12,17,21,22]. For the stimulation of guanylyl cyclase activity, crude membranes (10 μg of protein) were incubated with Mg^2+^ GTP and ATP (basal activity), 0.1–100 nm ANP, ATP and Mg^2+^ GTP (stimulated activity) or Triton X-100 and Mn^2+^ GTP (detergent-stimulated activity) for 10 min [12,17,21,22]. cGMP formation was measured by RIA [23]. Triton X-100 is known to maximally stimulate the receptor in a ligand-independent manner [12,17,21,22]. Therefore, the basal and ANP-stimulated cGMP responses were calculated as a percentage of the maximal Triton-stimulated activity. GC-A expression levels in the crude membranes were controlled by western blotting.

### Data analysis

Statistical comparisons were performed using Student’s *t*-test (*P* < 0.05). Data are given as the mean ± SE.

## Abbreviations

aa: amino acid
ANP: atrial natriuretic peptide
BNP: B-type natriuretic peptide
cGMP: guanosine 3′,5′-cyclic monophosphate
ERK: extracellular signal-regulated kinase
FRET: fluorescence resonance energy transfer
GC: guanylyl cyclase
HEK293: human embryonic kidney cell line
KH domain: kinase homology domain
LC: liquid chromatography
PMCA: plasma membrane-bound Ca^2+^-ATPase

## Supporting information

The following supplementary material is available:

**Fig. S1.** Sequence coverage of GC-A by MS.

**Fig. S2.** Fragment ion spectra of all identified tryptic GC-A phosphopeptides obtained from GC-A, exogenously expressed in HEK293 cells.

**Fig. S3.** Fragment ion spectra of all identified tryptic GC-A phosphopeptides obtained from GC-A, endogenously expressed in murine microvascular myocardial endothelial cells.

**Table S1.** Site-directed mutagenesis. Oligonucleotides and PCRs used to generate the plasmid for expression of GC-A S487E.

This supplementary material can be found in the online version of this article.

Please note: As a service to our authors and readers, this journal provides supporting information supplied by the authors. Such materials are peer-reviewed and may be re-organized for online delivery, but are not copy-edited or typeset. Technical support issues arising from supporting information (other than missing files) should be addressed to the authors.
