# Supplementary material for: Homologous desensitization of guanylyl cyclase A, the receptor for atrial natriuretic peptide, is associated with a complex phosphorylation pattern
Source: FEBS J. 2010 Jun;277(11):2440–53. doi: 10.1111/j.1742-4658.2010.07658.x (PMC2901513; doi:10.1111/j.1742-4658.2010.07658.x)
Supplement: Supplementary file 1 [file ejb0277-2440-SD1.pdf]

**Supplementary Fig. S1.** Sequence coverage of GC-A by mass spectrometry. The detected tryptic peptides, indicated in bold and underlined letters, covered 61 % of the GC-A sequence. Very dense sequence coverage was obtained at the N-terminus, within the kinase homology domain, and in the first part of the catalytic domain.

*signal sequence*

-28

MPGSRRVRPR LRALLLLPPL LLLRGGHASD LTVAVVLPLT NTSYPWSWAR

**VGPAVELALA** **R**VK**ARPDLLP** **GWTVRMVLGS** **SENAAGVCSD** **TAAPLAAVDL**

**KWEHSPAVFL** **GPGCVYSAAP** **VGRFTAHWRV** **PLLTAGAPAL** **GIGVKDEYAL**

**TTR**TGPSHVK **LGDFVTALHR** **RLGWEHQALV** **LYADRLGDDR** **PCFFIVEGLY**

**MR**VRER**LNIT** **VNHQEFVEGD** **PDHYPK**LLRA VRRKGR**VIYI** **CSSPDAFR**NL

MLLALNAGLT GEDYVFFHLD VFGQSLK**SAQ** **GLVPQKPWER** GDGQDRSARQ

AFQAAK**IITY** **KEPDNPEYLE** **FLK**QLKLLAD K**KFNFTVEDG** **LK**NIIPASFH

DGLLLYVQAV TETLAQGGTV TDGENITQRM WNR**SFQGVTG** **YLK**IDR**NGDR**

**DTDFSLWDMD** **PETGAFR**VVL NYNGTSQELM AVSEHK**LYWP** **LGYPPPDVPK**

CGFDNEDPAC NQDHFSTLEV LALVGSLSLI SFLIVSFFIY RKMQLEK**ELV**

**SELWRVRWED** **LQPSSLER**HL R**SAGSRLTLS** **GRGSNYGSLL** **TTEGQFQVFA**

**K**TAYYK**GNLV** **AVKR**VNRK**RI** **ELTRKVLFEL** **K**HMR**DVQNEH** **LTRFVGACTD**

**PPNICILTEY** **CPRGSLQDIL** **ENESITLDWM** **FRYSLTNDIV** **KGMLFLHNGA**

**ICSHGNLK**SS NCVVDGRFVL K**ITDYGLESF** **RDPEPEQGHT** **LFAKKLWTAP**

**ELLR**MASPPA R**GSQAGDVYS** **FGIILQEIAL** **RSGVFYVEGL** **DLSPK**EIIER

VTR**GEQPPFR** **PSMDLQSHLE** **ELGQLMQRCW** **AEDPQERPPF** **QQIR**LALRK**F**

**NKENSSNILD** **NLLSRMEQYA** **NNLEELVEER** **TQAYLEEKRK** **AEALLYQILP**

**HSVAEQLK**RG ETVQAEAFDS VTIYFSDIVG FTALSAESTP MQVVTLLNDL

YTCFDAVIDN FDVYK**VETIG** **DAYMVVSGLP** **VR**NGQLHARE VAR**MALALLD**

**AVR**SFRIRHR PQEQLRLR**IG** **IHTGPVCAGV** **VGLK**MPR**YCL** **FGDTVNTASR**

**MESNGEALKI** **HLSSETKAVL** **EEFDGFELEL** **R**GDVEMKGKG KVR**TYWLLGE**

**R**GCSTRG

1057

**Extracellular domain**

**Kinase homology domain**

**Catalytic cyclase domain**
