# Supplementary material for: Homologous desensitization of guanylyl cyclase A, the receptor for atrial natriuretic peptide, is associated with a complex phosphorylation pattern
Source: FEBS J. 2010 Jun;277(11):2440–53. doi: 10.1111/j.1742-4658.2010.07658.x (PMC2901513; doi:10.1111/j.1742-4658.2010.07658.x)
Supplement: Supplementary file 2 [file ejb0277-2440-SD2.pdf]

Supplementary Fig. S2. Fragment ion spectra of all identified tryptic GC-A phosphopeptides obtained from GC-A, exogenously expressed in HEK293 cells**.** Note that ion masses within the included tables can refer to intact b- and y-ions and to b- and y-ions after ß-elimination of phosphoric acid. *, neutral loss of NH_3; ^{0}, neutral loss of H_2O.

Mascot Search Results

Peptide View

MS/MS Fragmentation of **VRWEDLQPSSLER**
Found in **ANPRA_RAT**, Atrial natriuretic peptide receptor A precursor (ANP-A) (ANPRA) (GC-A) (Guanylate cyclase) (EC 4.6.1.2) (NPR-A) (Atrial natriuretic peptide A-type receptor) - Rattus norvegicus (Rat)

Match to Query 309: 1693.955382 from(565.659070,3+)
Title: File: QstarE03478.wiff, Sample: JS TiO2 Probe b (sample number 1), Elution: 91.442 to 91.528 min, Period: 1, Cycle(s): 9072-9074 (Experiment 2)
Data file C:\Dokumente und Einstellungen\Juliane\Eigene Dateien\QStar-Files\18042008\QstarE03478.mgf

Click mouse within plot area to zoom in by factor of two about that point
Or, Plot from 150 to 950 Da Full range

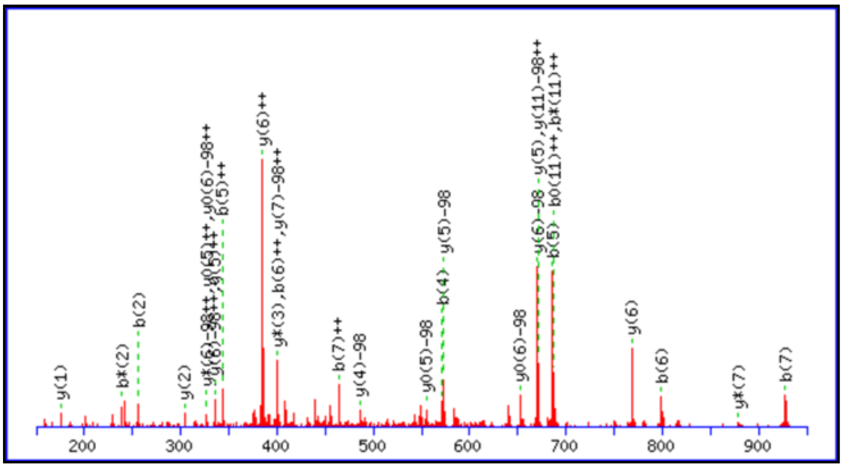

Monoisotopic mass of neutral peptide Mr(calc): 1693.7825
Fixed modifications: Carbamidomethyl (C)
Variable modifications:
S10 : Phospho (ST), with neutral losses 97.9769(shown in table), 0.0000
Ions Score: 37 **Expect:** 0.0075
Matches (Bold Red): 30/206 fragment ions using 35 most intense peaks

#	b	b^{++}	b*	b^{*++}	b^{0}	b^{0++}	Seq.	y	y^{++}	y*	y^{*++}	y^{0}	y^{0++}	#
1	100.0757	50.5415					V							13
2	**256.1768**	128.5920	**239.1503**	120.0788			R	1497.7445	749.3759	1480.7179	740.8626	1479.7339	740.3706	12
3	442.2561	221.6317	425.2296	213.1184			W	1341.6433	**671.3253**	1324.6168	662.8120	1323.6328	662.3200	11
4	**571.2987**	286.1530	554.2722	277.6397	553.2881	277.1477	E	1155.5640	578.2857	1138.5375	569.7724	1137.5535	569.2804	10
5	**686.3257**	**343.6665**	669.2991	335.1532	668.3151	334.6612	D	1026.5214	513.7644	1009.4949	505.2511	1008.5109	504.7591	9
6	**799.4097**	**400.2085**	782.3832	391.6952	781.3991	391.2032	L	911.4945	456.2509	894.4680	447.7376	893.4839	447.2456	8
7	**927.4683**	**464.2378**	910.4417	455.7245	909.4577	455.2325	Q	798.4104	**399.7089**	781.3839	391.1956	780.3999	390.7036	7
8	1024.5211	512.7642	1007.4945	504.2509	1006.5105	503.7589	P	**670.3519**	**335.6796**	653.3253	**327.1663**	**652.3413**	**326.6743**	6
9	1111.5531	556.2802	1094.5265	547.7669	1093.5425	547.2749	S	**573.2991**	287.1532	556.2725	278.6399	**555.2885**	278.1479	5
10	1180.5745	590.7909	1163.5480	582.2776	1162.5640	581.7856	S	**486.2671**	243.6372	469.2405	235.1239	468.2565	234.6319	4
11	1293.6586	647.3329	1276.6321	638.8197	1275.6480	638.3277	L	417.2456	209.1264	**400.2191**	200.6132	399.2350	200.1212	3
12	1422.7012	711.8542	1405.6746	703.3410	1404.6906	702.8490	E	**304.1615**	152.5844	287.1350	144.0711	286.1510	143.5791	2
13							R	**175.1190**	88.0631	158.0924	79.5498			1

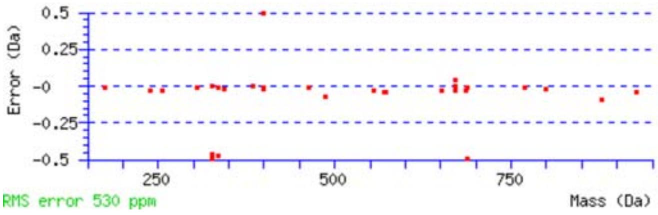

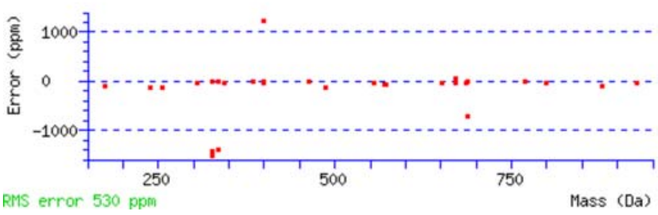

NCBI **BLAST** search of VRWEDLQPSSLER
(Parameters: blastp, nr protein database, expect=20000, no filter, PAM30)
Other BLAST web gateways

All matches to this query

Score	Mr(calc):	Delta	Sequence
36.7	1693.7825	0.1729	VRWEDLQPSSLER
30.6	1693.7825	0.1729	VRWEDLQPSSLER
9.6	1693.7560	0.1994	DSEEGGLSVAVPGEIR
8.5	1693.8049	0.1504	TSYAQHQQVRQIR
8.5	1693.8049	0.1504	TSYAQHQQVRQIR
6.2	1693.6936	0.2618	FKCPSSGTPSPTLR
6.0	1693.8049	0.1504	TSYAQHQQVRQIR
5.1	1693.6936	0.2618	FKCPSSGTPSPTLR
4.3	1693.7937	0.1617	DLGPRTQAWLQSSR
4.3	1693.7937	0.1617	DLGPRTQAWLQSSR

Mascot: http://www.matrixscience.com/

Mascot Search Results

Peptide View

MS/MS Fragmentation of **WEDLQPSSLER**
Found in **ANPRA_RAT**, Atrial natriuretic peptide receptor A precursor (ANP-A) (ANPRA) (GC-A) (Guanylate cyclase) (EC 4.6.1.2) (NPR-A) (Atrial natriuretic peptide A-type receptor) - Rattus norvegicus (Rat)

Match to Query 205: 1438.724278 from(720.369415,2+)
Title: File: QstarE03478.wiff, Sample: JS TiO2 Probe b (sample number 1), Elution: 83.36 to 83.436 min, Period: 1, Cycle(s): 8395-8397 (Experiment 2)
Data file C:\Dokumente und Einstellungen\Juliane\Eigene Dateien\QStar-Files\18042008\QstarE03478.mgf

Click mouse within plot area to zoom in by factor of two about that point
Or, Plot from 100 to 1400 Da Full range

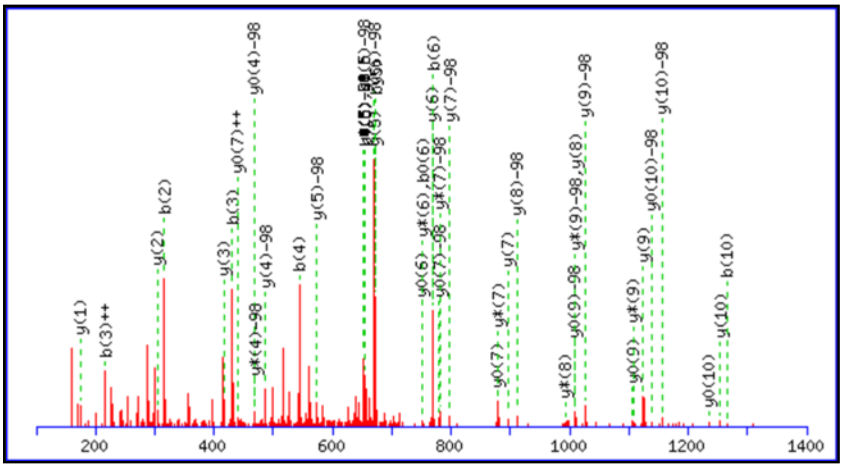

Monoisotopic mass of neutral peptide Mr(calc): 1438.6129
Fixed modifications: Carbamidomethyl (C)
Variable modifications:
S8 : Phospho (ST), with neutral losses 97.9769(shown in table), 0.0000
Ions Score: 39 **Expect:** 0.0039
Matches (Bold Red): 46/168 fragment ions using 116 most intense peaks

#	b	b^{++}	b*	b^{*++}	b^{0}	b^{0++}	Seq.	y	y^{++}	y*	y^{*++}	y^{0}	y^{0++}	#
1	187.0866	94.0469					W							11
2	**316.1292**	158.5682			298.1186	149.5629	E	**1155.5640**	578.2857	1138.5375	569.7724	**1137.5535**	569.2804	10
3	**431.1561**	**216.0817**			413.1456	207.0764	D	**1026.5214**	513.7644	**1009.4949**	505.2511	**1008.5109**	504.7591	9
4	**544.2402**	272.6237			526.2296	263.6185	L	**911.4945**	456.2509	894.4679	447.7376	893.4839	447.2456	8
5	**672.2988**	336.6530	**655.2722**	328.1397	**654.2882**	327.6477	Q	**798.4104**	399.7089	**781.3839**	391.1956	**780.3999**	390.7036	7
6	**769.3515**	385.1794	752.3250	376.6661	**751.3410**	376.1741	P	**670.3519**	335.6796	**653.3253**	327.1663	**652.3413**	326.6743	6
7	856.3836	428.6954	839.3570	420.1821	838.3730	419.6901	S	**573.2991**	287.1532	556.2725	278.6399	555.2885	278.1479	5
8	925.4050	463.2061	908.3785	454.6929	907.3945	454.2009	S	**486.2671**	243.6372	**469.2405**	235.1239	**468.2565**	234.6319	4
9	1038.4891	519.7482	1021.4625	511.2349	1020.4785	510.7429	L	**417.2456**	209.1264	400.2191	200.6132	399.2350	200.1212	3
10	1167.5317	584.2695	1150.5051	575.7562	1149.5211	575.2642	E	**304.1615**	152.5844	287.1350	144.0711	286.1510	143.5791	2
11							R	**175.1190**	88.0631	158.0924	79.5498			1

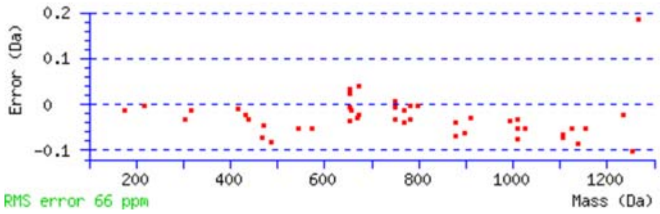

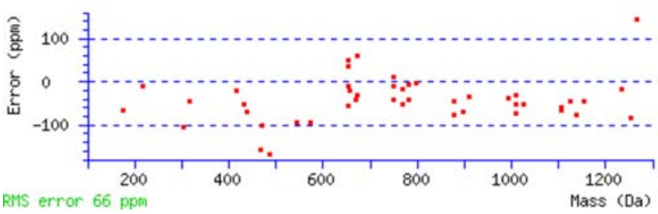

NCBI **BLAST** search of WEDLQPSSLER
(Parameters: blastp, nr protein database, expect=20000, no filter, PAM30)
Other BLAST web gateways

All matches to this query

Score	Mr(calc):	Delta	Sequence
39.2	1438.6129	0.1113	WEDLQPSSLER
31.5	1438.6129	0.1113	WEDLQPSSLER
8.0	1438.6929	0.0314	SSNTALLNREVR
6.6	1438.6453	0.0790	SGAQASSTPLSPTR
6.1	1438.6388	0.0855	MRGPGVGSGLSGER
5.8	1438.6453	0.0790	SGAQASSTPLSPTR
4.9	1438.7333	-0.0091	SSQQLLWTLKR
4.3	1438.6817	0.0425	SGGGSSNGLVGGLLGK
3.4	1438.7105	0.0137	HELYKAHEWAR
2.3	1438.6146	0.1097	VSESYAIIIER

Mascot: http://www.matrixscience.com/

Mascot Search Results

Peptide View

MS/MS Fragmentation of **SAGSRLTLSGR**
Found in **ANPRA_RAT**, Atrial natriuretic peptide receptor A precursor (ANP-A) (ANPRA) (GC-A) (Guanylate cyclase) (EC 4.6.1.2) (NPR-A) (Atrial natriuretic peptide A-type receptor) - Rattus norvegicus (Rat)

Match to Query 67: 1183.598172 from(395.540000,3+)
Title: File: Qtrap0014576.wiff, Sample: JS 080809 TiO2 (sample number 1), Elution: 31.983 min, Period: 1, Cycle(s): 832 (Experiment 4) (Charge not auto determined)
Data file C:\Dokumente und Einstellungen\Juliane\Eigene Dateien\Qtrap-files\2008\07042008\Qtrap0014576-1.mgf

Click mouse within plot area to zoom in by factor of two about that point
Or, Plot from 100 to 650 Da Full range

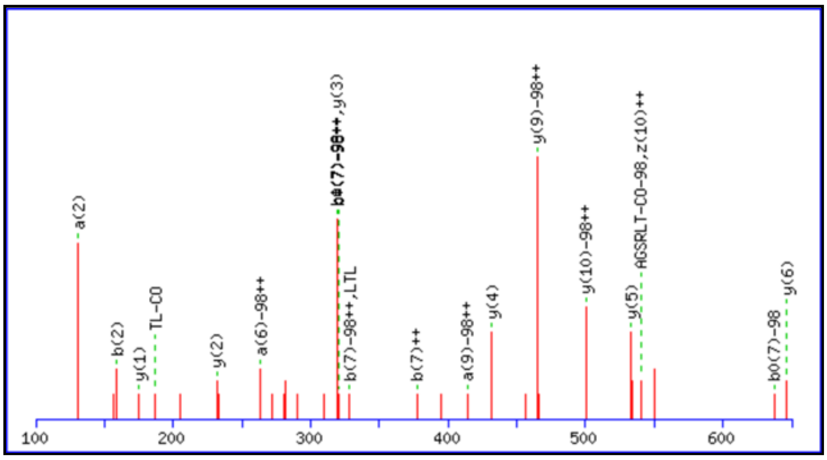

Monoisotopic mass of neutral peptide Mr(calc): 1183.5710
Fixed modifications: Carbamidomethyl (C)
Variable modifications:
S4 : Phospho (ST), with neutral losses 97.9769(shown in table), 0.0000
Ions Score: 41 **Expect:** 0.0029
Matches (Bold Red): 22/290 fragment ions using 25 most intense peaks

#	Immon.	a	a^{++}	b	b^{++}	b*	b^{*++}	b^0	b^{0++}	Seq.	y	y^{++}	z	z^{++}	#
1	60.0444	60.0444	30.5258	88.0393	44.5233			70.0287	35.5180	S					11
2	44.0495	**131.0815**	66.0444	**159.0764**	80.0418			141.0659	71.0366	A	999.5694	**500.2883**	982.5428	491.7751	10
3	30.0338	188.1030	94.5551	216.0979	108.5526			198.0873	99.5473	G	928.5323	**464.7698**	911.5057	456.2565	9
4	42.0338	257.1244	129.0658	285.1193	143.0633			267.1088	134.0580	S	871.5108	436.2590	854.4843	427.7458	8
5	129.1135	413.2255	207.1164	441.2204	221.1139	424.1939	212.6006	423.2099	212.1086	R	802.4894	401.7483	785.4628	393.2350	7
6	86.0964	526.3096	**263.6584**	554.3045	277.6559	537.2780	269.1426	536.2939	268.6506	L	**646.3883**	323.6978	629.3617	315.1845	6
7	74.0600	627.3573	314.1823	655.3522	**328.1797**	638.3256	**319.6665**	**637.3416**	**319.1745**	T	**533.3042**	267.1557	516.2776	258.6425	5
8	86.0964	740.4413	370.7243	768.4363	384.7218	751.4097	376.2085	750.4257	375.7165	L	**432.2565**	216.6319	415.2300	208.1186	4
9	60.0444	827.4734	**414.2403**	855.4683	428.2378	838.4417	419.7245	837.4577	419.2325	S	**319.1724**	160.0899	302.1459	151.5766	3
10	30.0338	884.4948	442.7511	912.4897	456.7485	895.4632	448.2352	894.4792	447.7432	G	**232.1404**	116.5738	215.1139	108.0606	2
11	129.1135									R	**175.1190**	88.0631	158.0924	79.5498	1

Seq	ya	yb	Seq	ya	yb	Seq	ya	yb
AG	101.0709	129.0659	AGS	170.0924	198.0873	AGSR	326.1935	354.1884
AGSRL	439.2776	467.2725	AGSRLT	**540.3252**	568.3202	AGSRLTL	653.4093	681.4042
GS	99.0553	127.0502	GSR	255.1564	283.1513	GSRL	368.2405	396.2354
GSRLT	469.2881	497.2831	GSRLTL	582.3722	610.3671	GSRLTLS	669.4042	697.3991
SR	198.1349	226.1298	SRL	311.2190	339.2139	SRLT	412.2667	440.2616
SRLTL	525.3507	553.3457	SRLTLS	612.3828	640.3777	SRLTLSG	669.4042	697.3991
RL	242.1975	270.1925	RLT	343.2452	371.2401	RLTL	456.3293	484.3242
RLTLS	543.3613	571.3562	RLTLSG	600.3828	628.3777	LT	**187.1441**	215.1390
LTL	300.2282	**328.2231**	LTLS	387.2602	415.2551	LTLSG	444.2817	472.2766
TL	**187.1441**	215.1390	TLS	274.1761	302.1710	TLSG	331.1976	359.1925
LS	173.1285	201.1234	LSG	230.1499	258.1448	SG	117.0659	145.0608

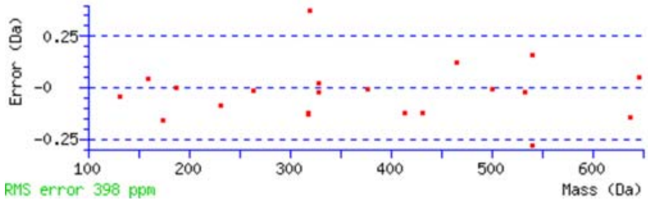

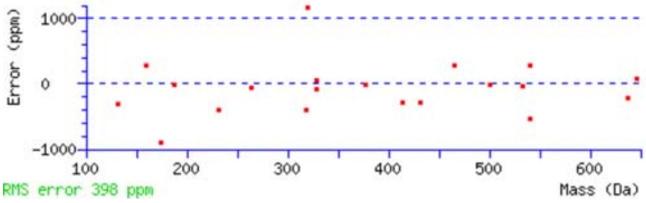

NCBI **BLAST** search of SAGSRLTLSGR
(Parameters: blastp, nr protein database, expect=20000, no filter, PAM30)
Other BLAST web gateways

All matches to this query

Score	Mr(calc):	Delta	Sequence
41.3	1183.5710	0.0271	SAGSRLTLSGR
35.9	1183.5710	0.0271	SAGSRLTLSGR
21.7	1183.5710	0.0271	SAGSRLTLSGR
16.2	1183.5784	0.0198	SACLRSLLGK
8.5	1183.5710	0.0271	SAGSRLTLSGR
7.9	1183.4750	0.1231	CPSSTWMGSR
7.7	1183.6244	-0.0262	DRMGTLAKHR
6.3	1183.5598	0.0384	LGASLGSLSSGR
5.8	1183.5122	0.0860	ALNSSAEDGIK
5.6	1183.5598	0.0384	LGASLGSLSSGR

Mascot: http://www.matrixscience.com/

Mascot Search Results

Peptide View

MS/MS Fragmentation of **SAGSRLTLSGR**
Found in **ANPRA_RAT**, Atrial natriuretic peptide receptor A precursor (ANP-A) (ANPRA) (GC-A) (Guanylate cyclase) (EC 4.6.1.2) (NPR-A) (Atrial natriuretic peptide A-type receptor) - Rattus norvegicus (Rat)

Match to Query 90: 1263.548172 from(422.190000,3+)
Title: File: Qtrap0014366.wiff, Sample: JS TiO2 2 (sample number 1), Elution: 22.769 to 22.882 min, Period: 1, Cycle(s): 521 (Experiment 3), 520 (Experiment 4) (Charge not auto determined)
Data file C:\Dokumente und Einstellungen\Juliane\Eigene Dateien\Qtrap-files\2008\20032008\Qtrap0014366-1.mgf

Click mouse within plot area to zoom in by factor of two about that point
Or, Plot from 100 to 800 Da Full range

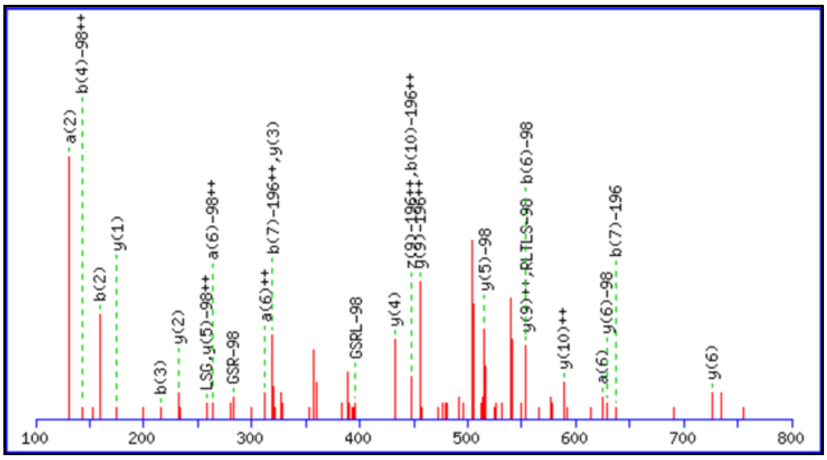

Monoisotopic mass of neutral peptide Mr(calc): 1263.5374
Fixed modifications: Carbamidomethyl (C)
Variable modifications:
S4 : Phospho (ST), with neutral losses 97.9769(shown in table), 0.0000
T7 : Phospho (ST), with neutral losses 97.9769(shown in table), 0.0000
Ions Score: 21 **Expect:** 0.065
Matches (Bold Red): 27/325 fragment ions using 48 most intense peaks

#	Immon.	a	a^{++}	b	b^{++}	b*	b^{*++}	b^0	b^{0++}	Seq.	y	y^{++}	z	z^{++}	#
1	60.0444	60.0444	30.5258	88.0393	44.5233			70.0287	35.5180	S					11
2	44.0495	**131.0815**	66.0444	**159.0764**	80.0418			141.0659	71.0366	A	981.5588	491.2830	964.5323	482.7698	10
3	30.0338	188.1030	94.5551	**216.0979**	108.5526			198.0873	99.5473	G	910.5217	**455.7645**	893.4952	**447.2512**	9
4	42.0338	257.1244	129.0658	285.1193	**143.0633**			267.1088	134.0580	S	853.5002	427.2538	836.4737	418.7405	8
5	129.1135	413.2255	207.1164	441.2204	221.1139	424.1939	212.6006	423.2099	212.1086	R	784.4788	392.7430	767.4522	384.2298	7
6	86.0964	526.3096	**263.6584**	**554.3045**	277.6559	537.2780	269.1426	536.2939	268.6506	L	**628.3777**	314.6925	611.3511	306.1792	6
7	56.0495	609.3467	305.1770	**637.3416**	**319.1744**	620.3151	310.6612	619.3311	310.1692	T	**515.2936**	**258.1504**	498.2671	249.6372	5
8	86.0964	722.4308	361.7190	750.4257	375.7165	733.3991	367.2032	732.4151	366.7112	L	**432.2565**	216.6319	415.2300	208.1186	4
9	60.0444	809.4628	405.2350	837.4577	419.2325	820.4312	410.7192	819.4471	410.2272	S	**319.1724**	160.0899	302.1459	151.5766	3
10	30.0338	866.4843	433.7458	894.4792	**447.7432**	877.4526	439.2300	876.4686	438.7379	G	**232.1404**	116.5738	215.1139	108.0606	2
11	129.1135									R	**175.1190**	88.0631	158.0924	79.5498	1

Seq	ya	yb	Seq	ya	yb	Seq	ya	yb
AG	101.0709	129.0659	AGS	170.0924	198.0873	AGSR	326.1935	354.1884
AGSRL	439.2776	467.2725	AGSRLT	522.3147	550.3096	AGSRLTL	635.3987	663.3937
GS	99.0553	127.0502	GSR	255.1564	**283.1513**	GSRL	368.2405	**396.2354**
GSRLT	451.2776	479.2725	GSRLTL	564.3616	592.3565	GSRLTLS	651.3937	679.3886
SR	198.1349	226.1298	SRL	311.2190	339.2139	SRLT	394.2561	422.2510
SRLTL	507.3402	535.3351	SRLTLS	594.3722	622.3671	SRLTLSG	651.3937	679.3886
RL	242.1975	270.1925	RLT	325.2346	353.2296	RLTL	438.3187	466.3136
RLTLS	525.3507	**553.3457**	RLTLSG	582.3722	610.3671	LT	169.1335	197.1284
LTL	282.2176	310.2125	LTLS	369.2496	397.2445	LTLSG	426.2711	454.2660
TL	169.1335	197.1284	TLS	256.1656	284.1605	TLSG	313.1870	341.1819
LS	173.1285	201.1234	LSG	230.1499	**258.1448**	SG	117.0659	145.0608

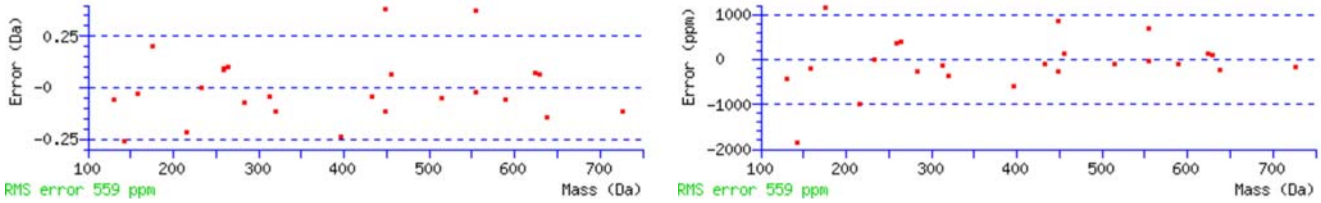

NCBI **BLAST** search of SAGSRLTLSGR
(Parameters: blastp, nr protein database, expect=20000, no filter, PAM30)
Other BLAST web gateways

All matches to this query

Score	Mr(calc):	Delta	Sequence
21.1	1263.5374	0.0108	SAGSRLTLSGR
18.0	1263.5374	0.0108	SAGSRLTLSGR
16.1	1263.5374	0.0108	SAGSRLTLSGR
9.1	1263.5261	0.0220	LGASLGSLSSGR
7.8	1263.5374	0.0108	SAGSRLTLSGR
6.5	1263.5374	0.0108	SAGSRLTLSGR
4.9	1263.5261	0.0220	LGASLGSLSSGR
4.4	1263.5261	0.0220	LGASLGSLSSGR
4.4	1263.5374	0.0108	SAGSRLTLSGR
3.5	1263.5860	-0.0379	GQSGLQPTGLLN

Mascot: http://www.matrixscience.com/

Mascot Search Results

Peptide View

MS/MS Fragmentation of **SAGSRLTLSGR**
Found in **ANPRA_RAT**, Atrial natriuretic peptide receptor A precursor (ANP-A) (ANPRA) (GC-A) (Guanylate cyclase) (EC 4.6.1.2) (NPR-A) (Atrial natriuretic peptide A-type receptor) - Rattus norvegicus (Rat)

Match to Query 106: 1263.642592 from(632.828572,2+)
Title: File: QstarE03481.wiff, Sample: JS IMAC/SIMAC E2b (sample number 1), Elution: 40.301 to 40.388 min, Period: 1, Cycle(s): 4426-4428 (Experiment 2)
Data file C:\Dokumente und Einstellungen\Juliane\Eigene Dateien\QStar-Files\18042008\QstarE03481.mgf

Click mouse within plot area to zoom in by factor of two about that point
Or, Plot from 150 to 1000 Da Full range

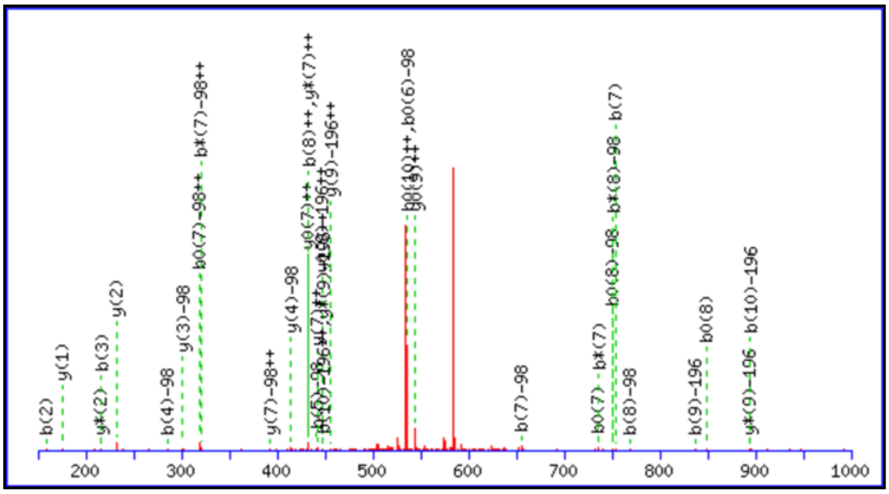

Monoisotopic mass of neutral peptide Mr(calc): 1263.5374
Fixed modifications: Carbamidomethyl (C)
Variable modifications:
S4 : Phospho (ST), with neutral losses 97.9769(shown in table), 0.0000
S9 : Phospho (ST), with neutral losses 97.9769(shown in table), 0.0000
Ions Score: 34 **Expect:** 0.012
Matches (Bold Red): 34/196 fragment ions using 69 most intense peaks

#	b	b^{++}	b*	b^{*++}	b^0	b^{0++}	Seq.	y	y^{++}	y*	y^{*++}	y^0	y^{0++}	#
1	88.0393	44.5233			70.0287	35.5180	S							11
2	**159.0764**	80.0418			141.0659	71.0366	A	981.5588	491.2830	964.5323	482.7698	963.5483	482.2778	10
3	**216.0979**	108.5526			198.0873	99.5473	G	910.5217	**455.7645**	**893.4952**	**447.2512**	892.5111	**446.7592**	9
4	**285.1193**	143.0633			267.1088	134.0580	S	853.5002	427.2538	836.4737	418.7405	835.4897	418.2485	8
5	**441.2204**	221.1139	424.1939	212.6006	423.2099	212.1086	R	784.4788	**392.7430**	767.4522	384.2298	766.4682	383.7378	7
6	554.3045	277.6559	537.2780	269.1426	**536.2939**	268.6506	L	628.3777	314.6925	611.3511	306.1792	610.3671	305.6872	6
7	**655.3522**	328.1797	638.3256	**319.6665**	637.3416	**319.1745**	T	515.2936	258.1504	498.2671	249.6372	497.2831	249.1452	5
8	**768.4363**	384.7218	**751.4097**	376.2085	**750.4257**	375.7165	L	**414.2459**	207.6266	397.2194	199.1133	396.2354	198.6213	4
9	**837.4577**	419.2325	820.4312	410.7192	819.4471	410.2272	S	**301.1619**	151.0846	284.1353	142.5713	283.1513	142.0793	3
10	**894.4792**	**447.7432**	877.4526	439.2300	876.4686	438.7379	G	**232.1404**	116.5738	**215.1139**	108.0606			2
11							R	**175.1190**	88.0631	158.0924	79.5498			1

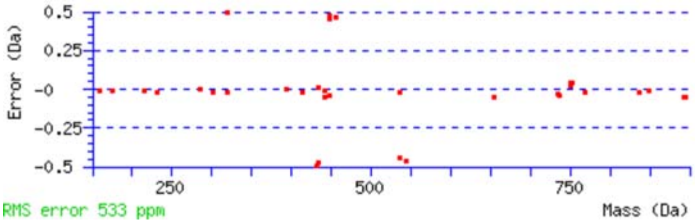

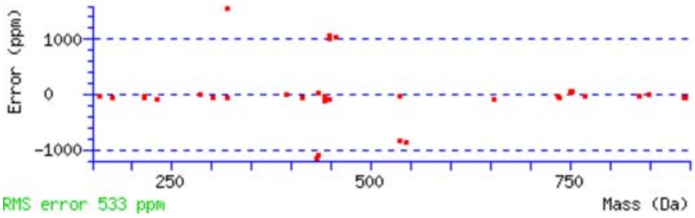

NCBI **BLAST** search of SAGSRLTLSGR
(Parameters: blastp, nr protein database, expect=20000, no filter, PAM30)
Other BLAST web gateways

All matches to this query

Score	Mr(calc):	Delta	Sequence
34.0	1263.5374	0.1052	SAGSRLTLSGR
28.3	1263.5374	0.1052	SAGSRLTLSGR
24.3	1263.5374	0.1052	SAGSRLTLSGR
17.8	1263.5374	0.1052	SAGSRLTLSGR
14.3	1263.5374	0.1052	SAGSRLTLSGR
14.3	1263.5374	0.1052	SAGSRLTLSGR
11.6	1263.5261	0.1165	TDKSAVSGAIR
11.6	1263.5261	0.1165	TDKSAVSGAIR
9.6	1263.5972	0.0453	SSGTHLEAKVR
8.7	1263.4414	0.2012	CPSSTWMGSR

Mascot: http://www.matrixscience.com/

Mascot Search Results

Peptide View

MS/MS Fragmentation of **SAGSRLTLSGR**
Found in **ANPRA_RAT**, Atrial natriuretic peptide receptor A precursor (ANP-A) (ANPRA) (GC-A) (Guanylate cyclase) (EC 4.6.1.2) (NPR-A) (Atrial natriuretic peptide A-type receptor) - Rattus norvegicus (Rat)

Match to Query 62: 1263.608172 from(422.210000,3+)
Title: File: Qtrap0014365.wiff, Sample: JS TiO2 1 (sample number 1), Elution: 20.433 to 20.548 min, Period: 1, Cycle(s): 514 (Experiment 3), 513 (Experiment 4) (Charge not auto determined)
Data file C:\Dokumente und Einstellungen\Juliane\Eigene Dateien\Qtrap-files\2008\20032008\Qtrap0014365-1.mgf

Click mouse within plot area to zoom in by factor of two about that point
Or, Plot from 100 to 800 Da Full range

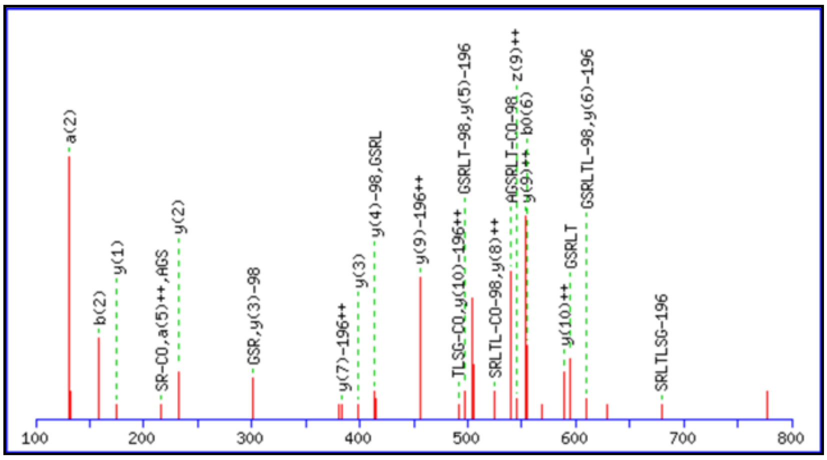

Monoisotopic mass of neutral peptide Mr(calc): 1263.5374
Fixed modifications: Carbamidomethyl (C)
Variable modifications:
T7 : Phospho (ST), with neutral losses 97.9769(shown in table), 0.0000
S9 : Phospho (ST), with neutral losses 97.9769(shown in table), 0.0000
Ions Score: 38 **Expect:** 0.0063
Matches (Bold Red): 31/301 fragment ions using 35 most intense peaks

#	Immon.	a	a^{++}	b	b^{++}	b*	b^{*++}	b^0	b^{0++}	Seq.	y	y^{++}	z	z^{++}	#
1	60.0444	60.0444	30.5258	88.0393	44.5233			70.0287	35.5180	S					11
2	44.0495	**131.0815**	66.0444	**159.0764**	80.0418			141.0659	71.0366	A	981.5588	**491.2830**	964.5323	482.7698	10
3	30.0338	188.1030	94.5551	**216.0979**	108.5526			198.0873	99.5473	G	910.5217	**455.7645**	893.4952	447.2512	9
4	60.0444	275.1350	138.0711	303.1299	152.0686			285.1193	143.0633	S	853.5002	427.2538	836.4737	418.7405	8
5	129.1135	431.2361	**216.1217**	459.2310	230.1191	442.2045	221.6059	441.2205	221.1139	R	766.4682	**383.7377**	749.4417	375.2245	7
6	86.0964	544.3202	272.6637	572.3151	286.6612	555.2885	278.1479	**554.3045**	277.6559	L	**610.3671**	305.6872	593.3406	297.1739	6
7	56.0495	627.3573	314.1823	655.3522	328.1797	638.3256	319.6665	637.3416	319.1745	T	**497.2830**	249.1452	480.2565	240.6319	5
8	86.0964	740.4413	370.7243	768.4363	384.7218	751.4097	376.2085	750.4257	375.7165	L	**414.2459**	207.6266	397.2194	199.1133	4
9	42.0338	809.4628	405.2350	837.4577	419.2325	820.4312	410.7192	819.4471	410.2272	S	**301.1619**	151.0846	284.1353	142.5713	3
10	30.0338	866.4843	433.7458	894.4792	447.7432	877.4526	439.2300	876.4686	438.7379	G	**232.1404**	116.5738	215.1139	108.0606	2
11	129.1135									R	**175.1190**	88.0631	158.0924	79.5498	1

Seq	ya	yb	Seq	ya	yb	Seq	ya	yb
AG	101.0709	129.0659	AGS	188.1030	**216.0979**	AGSR	344.2041	372.1990
AGSRL	457.2881	485.2831	AGSRLT	**540.3252**	568.3202	AGSRLTL	653.4093	681.4042
GS	117.0659	145.0608	GSR	273.1670	**301.1619**	GSRL	386.2510	**414.2459**
GSRLT	469.2881	**497.2831**	GSRLTL	582.3722	**610.3671**	GSRLTLS	651.3937	**679.3886**
SR	**216.1455**	244.1404	SRL	329.2296	357.2245	SRLT	412.2667	440.2616
SRLTL	**525.3507**	553.3457	SRLTLS	594.3722	622.3671	SRLTLSG	651.3937	**679.3886**
RL	242.1975	270.1925	RLT	325.2346	353.2296	RLTL	438.3187	466.3136
RLTLS	507.3402	535.3351	RLTLSG	564.3616	592.3565	LT	169.1335	197.1284
LTL	282.2176	310.2125	LTLS	351.2391	379.2340	LTLSG	408.2605	436.2554
TL	169.1335	197.1284	TLS	238.1550	266.1499	TLSG	295.1765	323.1714
LS	155.1179	183.1128	LSG	212.1393	240.1343	SG	99.0553	127.0502

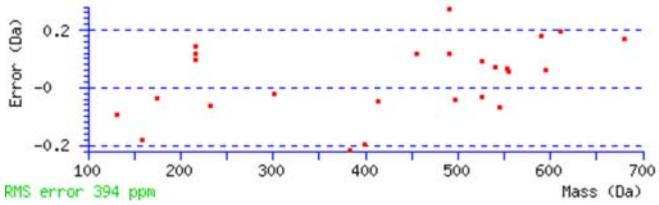

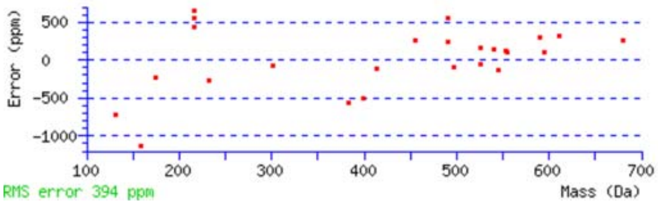

NCBI **BLAST** search of SAGSRLTLSGR
(Parameters: blastp, nr protein database, expect=20000, no filter, PAM30)
Other BLAST web gateways

All matches to this query

Score	Mr(calc):	Delta	Sequence
38.0	1263.5374	0.0708	SAGSRLTLSGR
27.0	1263.5374	0.0708	SAGSRLTLSGR
17.3	1263.5261	0.0820	LGASLGSLSSGR
11.2	1263.5261	0.0820	LGASLGSLSSGR
10.4	1263.5374	0.0708	SAGSRLTLSGR
9.7	1263.5261	0.0820	LGASLGSLSSGR
9.1	1263.5374	0.0708	SAGSRLTLSGR
8.4	1263.6700	-0.0619	GTRTQVPKGLK
8.3	1263.5036	0.1046	RRMMMQSGR
8.3	1263.5036	0.1046	RRMMMQSGR

Mascot: http://www.matrixscience.com/

Mascot Search Results

Peptide View

MS/MS Fragmentation of **SAGSRLTLSGR**
Found in **ANPRA_RAT**, Atrial natriuretic peptide receptor A precursor (ANP-A) (ANPRA) (GC-A) (Guanylate cyclase) (EC 4.6.1.2) (NPR-A) (Atrial natriuretic peptide A-type receptor) - Rattus norvegicus (Rat)

Match to Query 66: 1343.405448 from(672.710000,2+)
Title: File: Qtrap0014365.wiff, Sample: JS TiO2 1 (sample number 1), Elution: 28.005 to 28.118 min, Period: 1, Cycle(s): 681-682 (Experiment 3) (Charge not auto determined)
Data file C:\Dokumente und Einstellungen\Juliane\Eigene Dateien\Qtrap-files\2008\20032008\Qtrap0014365-1.mgf

Click mouse within plot area to zoom in by factor of two about that point
Or, Plot from 100 to 1000 Da Full range

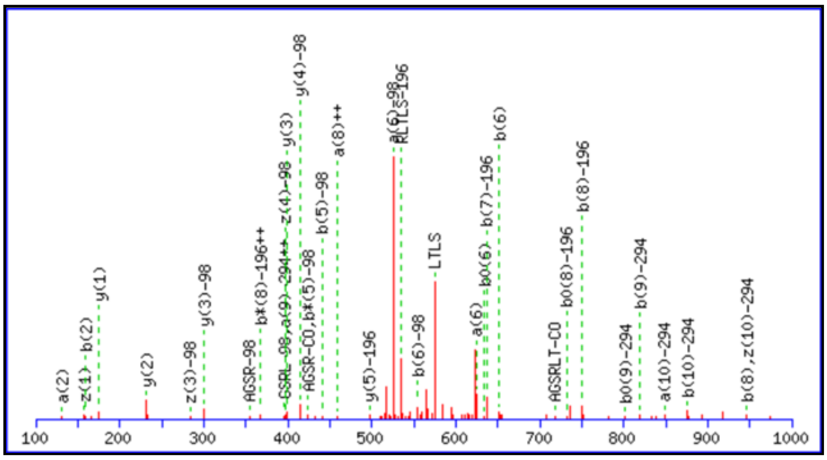


```
Monoisotopic mass of neutral peptide Mr(calc): 1343.5037
Fixed modifications: Carbamidomethyl (C)
Variable modifications:
S4     : Phospho (ST), with neutral losses 97.9769(shown in table), 0.0000
T7     : Phospho (ST), with neutral losses 97.9769(shown in table), 0.0000
S9     : Phospho (ST), with neutral losses 97.9769(shown in table), 0.0000
Ions Score: 32  Expect: 0.025
Matches (Bold Red): 37/342 fragment ions using 67 most intense peaks
```

#	Immon.	a	a^{++}	b	b^{++}	b*	b^{*++}	b^0	b^{0++}	Seq.	y	y^{++}	z	z^{++}	#
1	60.0444	60.0444	30.5258	88.0393	44.5233			70.0287	35.5180	S					11
2	44.0495	**131.0815**	66.0444	**159.0764**	80.0418			141.0659	71.0366	A	963.5483	482.2778	**946.5217**	473.7645	10
3	30.0338	188.1030	94.5551	216.0979	108.5526			198.0873	99.5473	G	892.5111	446.7592	875.4846	438.2459	9
4	42.0338	257.1244	129.0658	285.1193	143.0633			267.1088	134.0580	S	835.4897	418.2485	818.4631	409.7352	8
5	129.1135	413.2255	207.1164	**441.2204**	221.1139	**424.1939**	212.6006	423.2099	212.1086	R	766.4682	383.7377	749.4417	375.2245	7
6	86.0964	**526.3096**	263.6584	**554.3045**	277.6559	537.2780	269.1426	536.2939	268.6506	L	610.3671	305.6872	593.3406	297.1739	6
7	56.0495	609.3467	305.1770	**637.3416**	319.1744	620.3151	310.6612	619.3311	310.1692	T	**497.2830**	249.1452	480.2565	240.6319	5
8	86.0964	722.4308	361.7190	**750.4257**	375.7165	733.3991	**367.2032**	**732.4151**	366.7112	L	**414.2459**	207.6266	**397.2194**	199.1133	4
9	42.0338	791.4522	**396.2298**	**819.4471**	410.2272	802.4206	401.7139	**801.4366**	401.2219	S	**301.1619**	151.0846	**284.1353**	142.5713	3
10	30.0338	**848.4737**	424.7405	**876.4686**	438.7379	859.4421	430.2247	858.4580	429.7327	G	**232.1404**	116.5738	215.1139	108.0606	2
11	129.1135									R	**175.1190**	88.0631	**158.0924**	79.5498	1

Seq	ya	yb	Seq	ya	yb	Seq	ya	yb
AG	101.0709	129.0659	AGS	170.0924	198.0873	AGSR	326.1935	**354.1884**
AGSRL	439.2776	467.2725	AGSRLT	522.3147	550.3096	AGSRLTL	635.3987	663.3937
GS	99.0553	127.0502	GSR	255.1564	283.1513	GSRL	368.2405	**396.2354**
GSRLT	451.2776	479.2725	GSRLTL	564.3616	592.3565	GSRLTLS	633.3831	661.3780
GSRLTLSG	690.4045	718.3995	SR	198.1349	226.1298	SRL	311.2190	339.2139
SRLT	394.2561	422.2510	SRLTL	507.3402	**535.3351**	SRLTLS	576.3616	604.3565
SRLTLSG	633.3831	661.3780	RL	242.1975	270.1925	RLT	325.2346	353.2296
RLTL	438.3187	466.3136	RLTLS	507.3402	**535.3351**	RLTLSG	564.3616	592.3565
LT	169.1335	197.1284	LTL	282.2176	310.2125	LTLS	351.2391	379.2340
LTLSG	408.2605	436.2554	TL	169.1335	197.1284	TLS	238.1550	266.1499
TLSG	295.1765	323.1714	LS	155.1179	183.1128	LSG	212.1393	240.1343

**SG**	99.0553	127.0502						

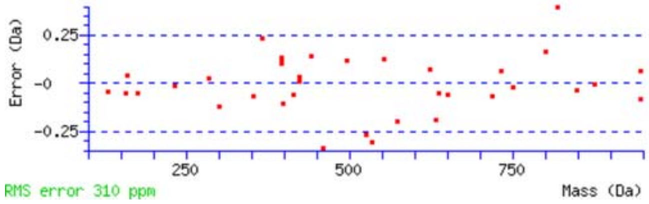

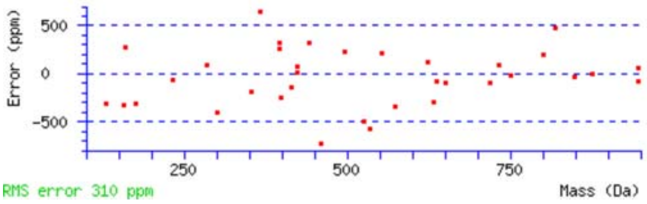

NCBI **BLAST** search of SAGSRLTLSGR
(Parameters: blastp, nr protein database, expect=20000, no filter, PAM30)
Other BLAST web gateways

All matches to this query

Score	Mr(calc):	Delta	Sequence
32.2	1343.5037	-0.0982	SAGSRLTLSGR
22.2	1343.5037	-0.0982	SAGSRLTLSGR
18.0	1343.5037	-0.0982	SAGSRLTLSGR
13.6	1343.4924	-0.0870	LGASLGSLSSGR
12.4	1343.4597	-0.0542	DNSTMGYMMAK
12.4	1343.4597	-0.0542	DNSTMGYMMAK
10.4	1343.4597	-0.0542	DNSTMGYMMAK
10.1	1343.5312	-0.1258	IPANWTNPSGK
9.8	1343.4496	-0.0441	APTTVRCSGR
9.8	1343.4924	-0.0870	LGASLGSLSSGR

Mascot: http://www.matrixscience.com/

Mascot Search Results

Peptide View

MS/MS Fragmentation of **LTLSGR**
Found in **ANPRA_RAT**, Atrial natriuretic peptide receptor A precursor (ANP-A) (ANPRA) (GC-A) (Guanylate cyclase) (EC 4.6.1.2) (NPR-A) (Atrial natriuretic peptide A-type receptor) - Rattus norvegicus (Rat)

Match to Query 2: 725.345448 from(363.680000,2+)
Title: File: Qtrap0014365.wiff, Sample: JS TiO2 1 (sample number 1), Elution: 19.539 to 20.271 min, Period: 1, Cycle(s): 504-505, 511 (Experiment 3), 512 (Experiment 4) (Charge not auto determined)
Data file C:\Dokumente und Einstellungen\Juliane\Eigene Dateien\Qtrap-files\2008\20032008\Qtrap0014365-1.mgf

Click mouse within plot area to zoom in by factor of two about that point
Or, Plot from 120 to 620 Da Full range

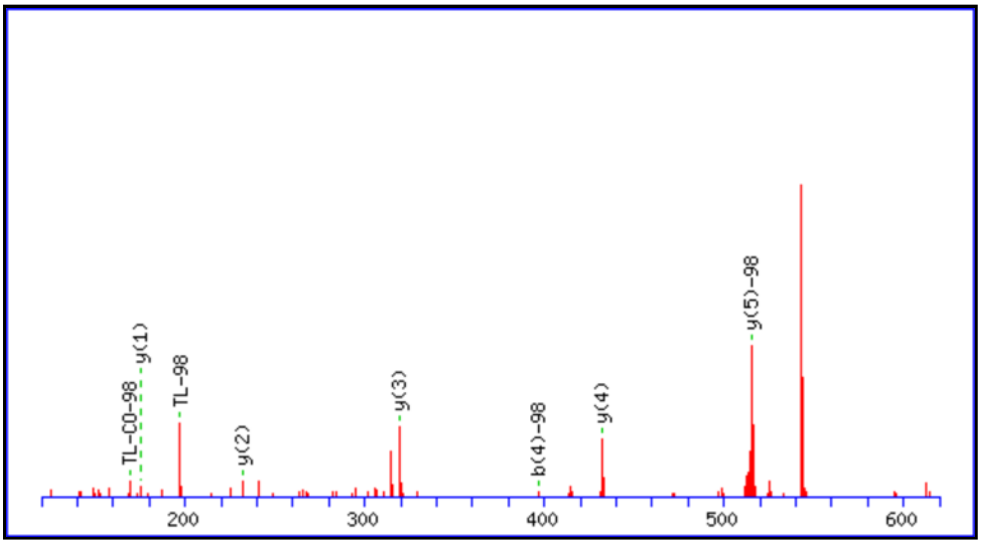

Monoisotopic mass of neutral peptide Mr(calc): 725.3473
Fixed modifications: Carbamidomethyl (C)
Variable modifications:
T2 : Phospho (ST), with neutral losses 97.9769(shown in table), 0.0000
Ions Score: 34 **Expect:** 0.011
Matches (Bold Red): 10/101 fragment ions using 17 most intense peaks

#	Immon.	a	a^{++}	b	b^{++}	b^0	b^{0++}	Seq.	y	y^{++}	z	z^{++}	#
1	86.0964	86.0964	43.5519	114.0913	57.5493			L					6
2	56.0495	**169.1335**	85.0704	**197.1284**	99.0679	179.1179	90.0626	T	**515.2936**	258.1504	498.2671	249.6372	5
3	86.0964	282.2176	141.6124	310.2125	155.6099	292.2019	146.6046	L	**432.2565**	216.6319	415.2300	208.1186	4
4	60.0444	369.2496	185.1284	**397.2445**	199.1259	379.2340	190.1206	S	**319.1724**	160.0899	302.1459	151.5766	3
5	30.0338	426.2711	213.6392	454.2660	227.6366	436.2554	218.6314	G	**232.1404**	116.5738	215.1139	108.0606	2
6	129.1135							R	**175.1190**	88.0631	158.0924	79.5498	1

Seq	ya	yb	Seq	ya	yb	Seq	ya	yb
TL	**169.1335**	**197.1284**	TLS	256.1656	284.1605	TLSG	313.1870	341.1819
LS	173.1285	201.1234	LSG	230.1499	258.1448	SG	117.0659	145.0608

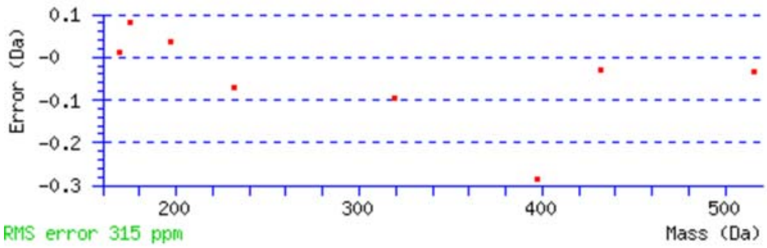

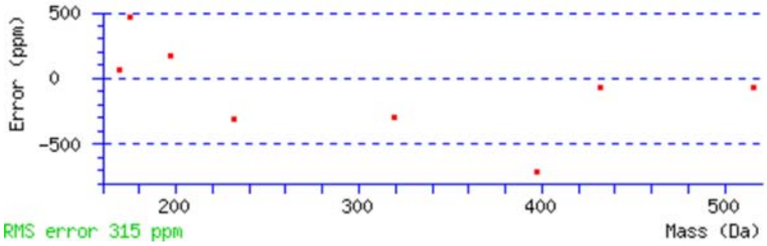

NCBI **BLAST** search of LTLSGR
(Parameters: blastp, nr protein database, expect=20000, no filter, PAM30)
Other BLAST web gateways

All matches to this query

Score	Mr(calc):	Delta	Sequence
33.5	725.3473	-0.0018	LTLSGR
12.2	725.3473	-0.0018	LTLSGR
8.0	725.3361	0.0094	ILTGDK
8.0	725.3088	0.0367	MIMDGK
5.7	725.3473	-0.0019	ITGTVR
5.6	725.2858	0.0597	NAAGTGR
4.0	725.3681	-0.0227	RNHSGR
3.6	725.3473	-0.0019	ITGTVR
3.3	725.2745	0.0709	TEPSGR
3.1	725.3725	-0.0270	LTVSVK

Mascot: http://www.matrixscience.com/

Mascot Search Results

Peptide View

MS/MS Fragmentation of **LTLSGR**
Found in **ANPRA_RAT**, Atrial natriuretic peptide receptor A precursor (ANP-A) (ANPRA) (GC-A) (Guanylate cyclase) (EC 4.6.1.2) (NPR-A) (Atrial natriuretic peptide A-type receptor) - Rattus norvegicus (Rat)

Match to Query 3: 725.345448 from(363.680000,2+)
Title: File: Qtrap0014365.wiff, Sample: JS TiO2 1 (sample number 1), Elution: 18.84 to 18.957 min, Period: 1, Cycle(s): 489-490 (Experiment 3) (Charge not auto determined)
Data file C:\Dokumente und Einstellungen\Juliane\Eigene Dateien\Qtrap-files\2008\20032008\Qtrap0014365-1.mgf

Click mouse within plot area to zoom in by factor of two about that point
Or, Plot from 100 to 650 Da Full range

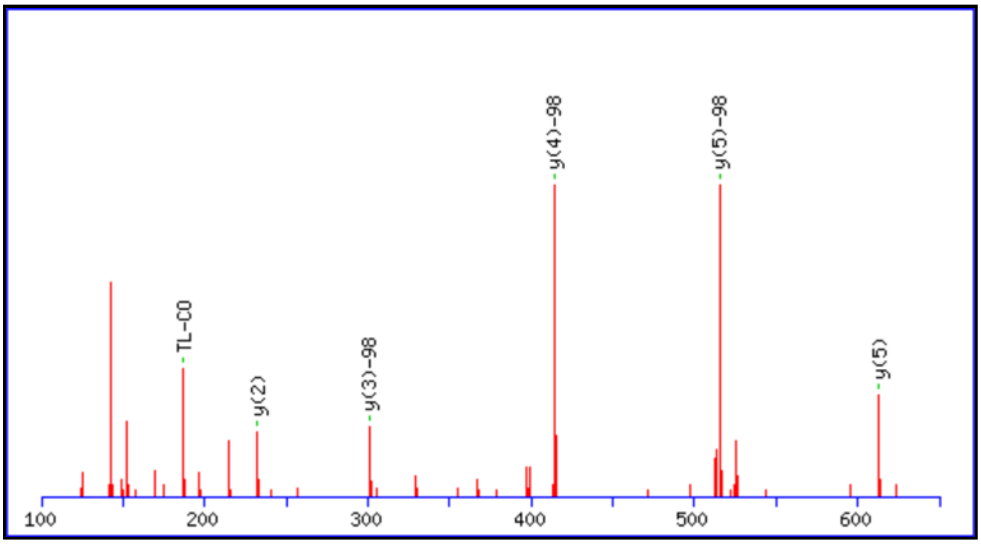

Monoisotopic mass of neutral peptide Mr(calc): 725.3473
Fixed modifications: Carbamidomethyl (C)
Variable modifications:
S4 : Phospho (ST), with neutral losses 97.9769(shown in table), 0.0000
Ions Score: 28 **Expect:** 0.037
Matches (Bold Red): 7/101 fragment ions using 10 most intense peaks

#	Immon.	a	a^{++}	b	b^{++}	b^0	b^{0++}	Seq.	y	y^{++}	z	z^{++}	#
1	86.0964	86.0964	43.5519	114.0913	57.5493			L					6
2	74.0600	**187.1441**	94.0757	215.1390	108.0731	197.1285	99.0679	T	**515.2936**	258.1504	498.2671	249.6372	5
3	86.0964	300.2282	150.6177	328.2231	164.6152	310.2125	155.6099	L	**414.2459**	207.6266	397.2194	199.1133	4
4	42.0338	369.2496	185.1284	397.2445	199.1259	379.2340	190.1206	S	**301.1619**	151.0846	284.1353	142.5713	3
5	30.0338	426.2711	213.6392	454.2660	227.6366	436.2554	218.6314	G	**232.1404**	116.5738	215.1139	108.0606	2
6	129.1135							R	175.1190	88.0631	158.0924	79.5498	1

Seq	ya	yb	Seq	ya	yb	Seq	ya	yb
TL	**187.1441**	215.1390	TLS	256.1656	284.1605	TLSG	313.1870	341.1819
LS	155.1179	183.1128	LSG	212.1393	240.1343	SG	99.0553	127.0502

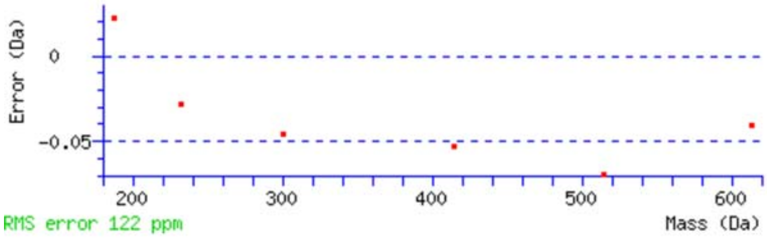

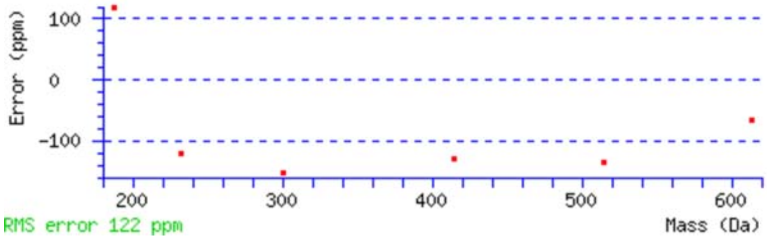

NCBI **BLAST** search of LTLSGR
(Parameters: blastp, nr protein database, expect=20000, no filter, PAM30)
Other BLAST web gateways

All matches to this query

Score	Mr(calc):	Delta	Sequence
28.0	725.3473	-0.0018	LTLSGR
10.5	725.3109	0.0345	TNLNGK
9.6	725.3473	-0.0019	ITGTVR
9.6	725.3725	-0.0270	LTVSVK
7.9	725.3473	-0.0018	LTLSGR
7.8	725.3361	0.0094	LDVTAK
6.2	725.4184	-0.0730	ITSLHR
4.4	725.4072	-0.0617	LSEIHK
3.3	725.4072	-0.0617	LTLKHD
1.4	725.3473	-0.0018	ISKNGK

Mascot: http://www.matrixscience.com/

Mascot Search Results

Peptide View

MS/MS Fragmentation of **LTLSGR**
Found in **ANPRA_RAT**, Atrial natriuretic peptide receptor A precursor (ANP-A) (ANPRA) (GC-A) (Guanylate cyclase) (EC 4.6.1.2) (NPR-A) (Atrial natriuretic peptide A-type receptor) - Rattus norvegicus (Rat)

Match to Query 22: 805.245448 from(403.630000,2+)
Title: File: Qtrap0014108.wiff, Sample: JS TiO2 1 (sample number 1), Elution: 25.559 min, Period: 1, Cycle(s): 201 (Experiment 3) (Charge not auto determined)
Data file C:\Dokumente und Einstellungen\Juliane\Eigene Dateien\Qtrap-files\2008\22022008\Qtrap0014108-1.mgf

Click mouse within plot area to zoom in by factor of two about that point
Or, Plot from 150 to 700 Da Full range

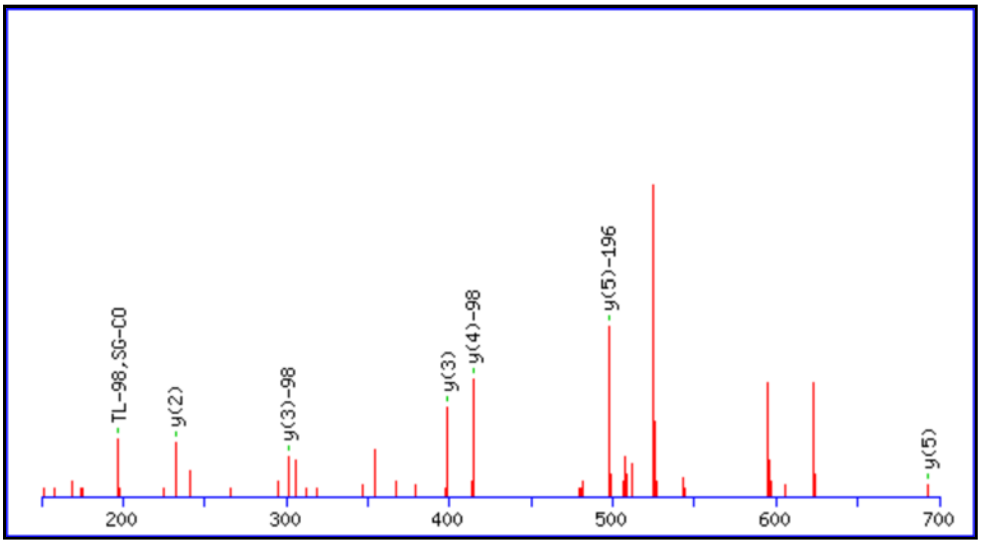

Monoisotopic mass of neutral peptide Mr(calc): 805.3136
Fixed modifications: Carbamidomethyl (C)
Variable modifications:
T2 : Phospho (ST), with neutral losses 97.9769(shown in table), 0.0000
S4 : Phospho (ST), with neutral losses 97.9769(shown in table), 0.0000
Ions Score: 26 **Expect:** 0.013
Matches (Bold Red): 9/116 fragment ions using 13 most intense peaks

#	Immon.	a	a^{++}	b	b^{++}	b^{0}	b^{0++}	Seq.	y	y^{++}	z	z^{++}	#
1	86.0964	86.0964	43.5519	114.0913	57.5493			L					6
2	56.0495	169.1335	85.0704	**197.1284**	99.0679	179.1179	90.0626	T	**497.2830**	249.1452	480.2565	240.6319	5
3	86.0964	282.2176	141.6124	310.2125	155.6099	292.2019	146.6046	L	**414.2459**	207.6266	397.2194	199.1133	4
4	42.0338	351.2391	176.1232	379.2340	190.1206	361.2234	181.1153	S	**301.1619**	151.0846	284.1353	142.5713	3
5	30.0338	408.2605	204.6339	436.2554	218.6314	418.2449	209.6261	G	**232.1404**	116.5738	215.1139	108.0606	2
6	129.1135							R	175.1190	88.0631	158.0924	79.5498	1

Seq	ya	yb	Seq	ya	yb	Seq	ya	yb
TL	169.1335	**197.1284**	TLS	238.1550	266.1499	TLSG	295.1765	323.1714
LS	155.1179	183.1128	LSG	212.1393	240.1343	SG	99.0553	127.0502

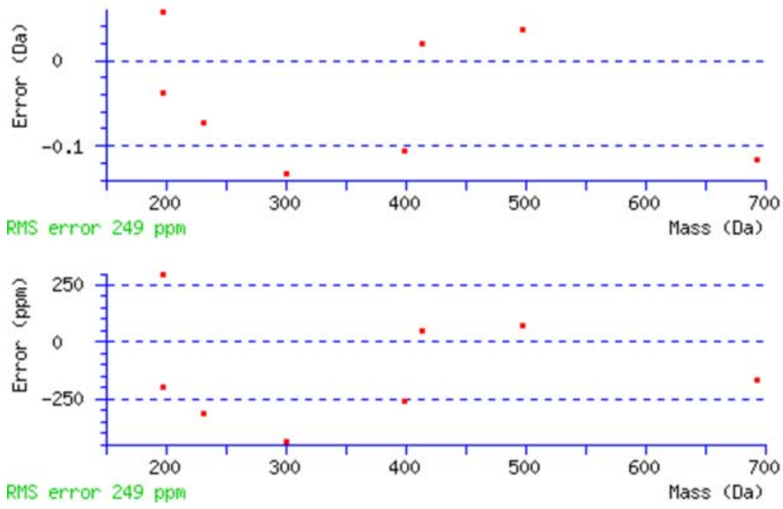

NCBI **BLAST** search of LTLSGR
(Parameters: blastp, nr protein database, expect=20000, no filter, PAM30)
Other BLAST web gateways

All matches to this query

Score	Mr(calc):	Delta	Sequence
25.7	805.3136	-0.0682	LTLSGR
2.9	805.3136	-0.0682	ITGTVR
1.4	805.2408	0.0046	TEPSGR
1.0	805.3136	-0.0682	LLSGTR

Mascot: http://www.matrixscience.com/

Mascot Search Results

Peptide View

MS/MS Fragmentation of **GSNYGSLLTTEGQFQVFAK**
Found in **ANPRA_RAT**, Atrial natriuretic peptide receptor A precursor (ANP-A) (ANPRA) (GC-A) (Guanylate cyclase) (EC 4.6.1.2) (NPR-A) (Atrial natriuretic peptide A-type receptor) - Rattus norvegicus (Rat)

Match to Query 207: 2125.765448 from(1063.890000,2+)
Title: File: Qtrap0014366.wiff, Sample: JS TiO2 2 (sample number 1), Elution: 61.581 min, Period: 1, Cycle(s): 1117 (Experiment 4) (Charge not auto determined)
Data file C:\Dokumente und Einstellungen\Juliane\Eigene Dateien\Qtrap-files\2008\20032008\Qtrap0014366-1.mgf

Click mouse within plot area to zoom in by factor of two about that point
Or, Plot from 100 to 1700 Da Full range

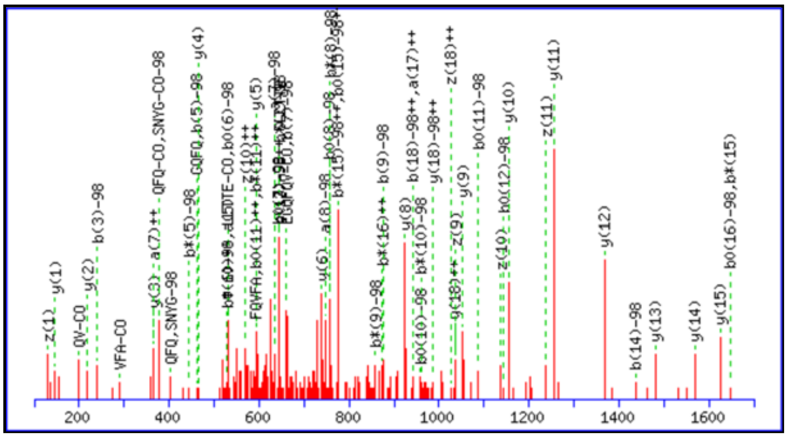

Monoisotopic mass of neutral peptide Mr(calc): 2125.9722
Fixed modifications: Carbamidomethyl (C)
Variable modifications:
S2 : Phospho (ST), with neutral losses 97.9769(shown in table), 0.0000
Ions Score: 84 **Expect:** 2e-007
Matches (Bold Red): 68/520 fragment ions using 101 most intense peaks

#	Immon.	a	a^{++}	b	b^{++}	b*	b^{*++}	b^{0}	b^{0++}	Seq.	y	y^{++}	z	z^{++}	#
1	30.0338	30.0338	15.5206	58.0287	29.5180					G					19
2	42.0338	99.0553	50.0313	127.0502	64.0287			109.0396	55.0235	S	1971.9811	**986.4942**	1954.9545	977.9809	18
3	87.0553	213.0982	107.0527	**241.0931**	121.0502	224.0666	112.5369	223.0826	112.0449	N	1902.9596	951.9834	1885.9331	943.4702	17
4	136.0757	**376.1615**	188.5844	**404.1565**	202.5819	387.1299	194.0686	386.1459	193.5766	Y	1788.9167	894.9620	1771.8901	886.4487	16
5	30.0338	433.1830	217.0951	**461.1779**	231.0926	**444.1514**	222.5793	443.1674	222.0873	G	**1625.8534**	813.4303	1608.8268	804.9170	15
6	60.0444	520.2150	260.6112	548.2099	274.6086	**531.1834**	266.0953	**530.1994**	265.6033	S	**1568.8319**	784.9196	1551.8053	776.4063	14
7	86.0964	**633.2991**	317.1532	**661.2940**	331.1506	**644.2675**	322.6374	**643.2834**	322.1454	L	**1481.7999**	741.4036	1464.7733	732.8903	13
8	86.0964	**746.3832**	373.6952	**774.3781**	387.6927	**757.3515**	379.1794	**756.3675**	378.6874	L	**1368.7158**	684.8615	1351.6892	676.3483	12
9	74.0600	847.4308	424.2191	**875.4258**	438.2165	**858.3992**	429.7032	857.4152	429.2112	T	**1255.6317**	628.3195	**1238.6052**	619.8062	11
10	74.0600	948.4785	474.7429	976.4734	488.7404	**959.4469**	480.2271	**958.4629**	479.7351	T	**1154.5841**	577.7957	**1137.5575**	**569.2824**	10
11	102.0550	1077.5211	539.2642	1105.5160	553.2616	1088.4895	544.7484	**1087.5055**	544.2564	E	**1053.5364**	527.2718	**1036.5098**	518.7586	9
12	30.0338	1134.5426	567.7749	1162.5375	581.7724	1145.5109	573.2591	**1144.5269**	572.7671	G	**924.4938**	462.7505	907.4672	454.2373	8
13	101.0709	1262.6012	631.8042	1290.5961	**645.8017**	1273.5695	637.2884	1272.5855	636.7964	Q	867.4723	434.2398	850.4458	425.7265	7
14	120.0808	1409.6696	705.3384	**1437.6645**	719.3359	1420.6379	710.8226	1419.6539	710.3306	F	**739.4137**	370.2105	722.3872	361.6972	6
15	101.0709	1537.7281	769.3677	1565.7231	783.3652	1548.6965	**774.8519**	1547.7125	**774.3599**	Q	**592.3453**	296.6763	575.3188	288.1630	5
16	72.0808	1636.7966	818.9019	1664.7915	832.8994	1647.7649	824.3861	**1646.7809**	823.8941	V	**464.2867**	232.6470	447.2602	224.1337	4
17	120.0808	1783.8650	892.4361	1811.8599	906.4336	1794.8333	897.9203	1793.8493	897.4283	F	**365.2183**	183.1128	348.1918	174.5995	3
18	44.0495	1854.9021	927.9547	1882.8970	**941.9521**	1865.8705	933.4389	1864.8864	932.9469	A	**218.1499**	109.5786	201.1234	101.0653	2
19	101.1073									K	**147.1128**	74.0600	**130.0863**	65.5468	1

Seq	ya	yb	Seq	ya	yb	Seq	ya	yb
SN	156.0767	184.0717	SNY	319.1401	347.1350	SNYG	**376.1615**	**404.1565**
SNYGS	463.1936	491.1885	SNYGSL	576.2776	604.2725	SNYGSLL	689.3617	717.3566
NY	250.1186	278.1135	NYG	307.1401	335.1350	NYGS	394.1721	422.1670
NYGSL	507.2562	535.2511	NYGSLL	620.3402	648.3352	YG	193.0972	221.0921
YGS	280.1292	308.1241	YGSL	393.2132	421.2082	YGSLL	506.2973	534.2922
YGSLLT	607.3450	635.3399	GS	117.0659	145.0608	GSL	230.1499	258.1448
GSLL	343.2340	371.2289	GSLLT	444.2817	472.2766	GSLLTT	545.3293	573.3243

**GSLLTTE**	674.3719	702.3668	**SL**	173.1285	201.1234	**SLL**	286.2125	314.2074
**SLLT**	387.2602	415.2551	**SLLTT**	488.3079	516.3028	**SLLTTE**	617.3505	**645.3454**
**SLLTTEG**	674.3719	702.3668	**LL**	199.1805	227.1754	**LLT**	300.2282	328.2231
**LLTT**	401.2758	429.2708	**LLTTE**	**530.3184**	558.3134	**LLTTEG**	587.3399	615.3348
**LT**	187.1441	215.1390	**LTT**	288.1918	316.1867	**LTTE**	417.2344	445.2293
**LTTEG**	474.2558	502.2508	**LTTEGQ**	602.3144	630.3093	**TT**	175.1077	203.1026
**TTE**	304.1503	332.1452	**TTEG**	361.1718	389.1667	**TTEGQ**	489.2304	517.2253
**TTEGQF**	636.2988	664.2937	**TE**	203.1026	231.0975	**TEG**	260.1241	288.1190
**TEGQ**	388.1827	416.1776	**TEGQF**	535.2511	563.2460	**TEGQFQ**	663.3097	691.3046
**EG**	159.0764	187.0713	**EGQ**	287.1350	315.1299	**EGQF**	434.2034	462.1983
**EGQFQ**	562.2620	590.2569	**EGQFQV**	**661.3304**	689.3253	**GQ**	158.0924	186.0873
**GQF**	305.1608	333.1557	**GQFQ**	433.2194	**461.2143**	**GQFQV**	532.2878	560.2827
**GQFQVF**	679.3562	707.3511	**QF**	248.1394	276.1343	**QFQ**	**376.1979**	**404.1928**
**QFQV**	475.2663	503.2613	**QFQVF**	622.3348	650.3297	**QFQVFA**	693.3719	721.3668
**FQ**	248.1394	276.1343	**FQV**	347.2078	375.2027	**FQVF**	494.2762	522.2711
**FQVFA**	565.3133	**593.3082**	**QV**	**200.1394**	228.1343	**QVF**	347.2078	375.2027
**QVFA**	418.2449	446.2398	**VF**	219.1492	247.1441	**VFA**	**290.1863**	318.1812
**FA**	191.1179	219.1128						

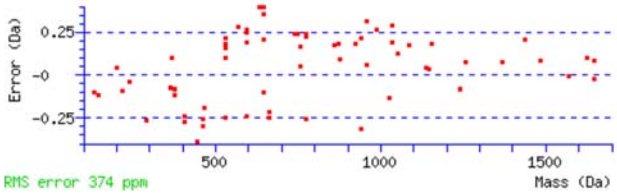

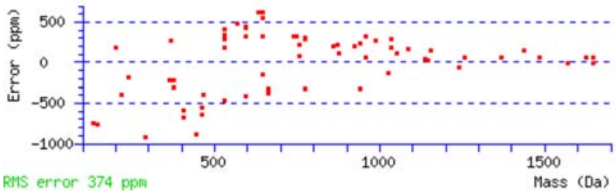

NCBI **BLAST** search of GSNYGSLLTTEGQFQVFAK
(Parameters: blastp, nr protein database, expect=20000, no filter, PAM30)
Other BLAST web gateways

All matches to this query

Score	Mr(calc):	Delta	Sequence
84.2	2125.9722	-0.2067	GSNYGSLLTTEGQFQVFAK
74.7	2125.9722	-0.2067	GSNYGSLLTTEGQFQVFAK
58.8	2125.9722	-0.2067	GSNYGSLLTTEGQFQVFAK
47.7	2125.9722	-0.2067	GSNYGSLLTTEGQFQVFAK
38.5	2125.9722	-0.2067	GSNYGSLLTTEGQFQVFAK
11.2	2125.8645	-0.0991	CGAPGACRMYDINSFRR
8.2	2125.8906	-0.1252	YGVYEAIFTMLSSLMNK
7.5	2125.8906	-0.1252	YGVYEAIFTMLSSLMNK
7.2	2125.8687	-0.1032	DSKMTRILQDSLGGNCR
6.3	2125.7921	-0.0266	EVAMTEHKMSVEEVCR

Mascot: http://www.matrixscience.com/

{MATRIX SCIENCE} Mascot Search Results

Peptide View

MS/MS Fragmentation of **GSNYGSLLTTEGQFQVFAK**
Found in **ANPRA_RAT**, Atrial natriuretic peptide receptor A precursor (ANP-A) (ANPRA) (GC-A) (Guanylate cyclase) (EC 4.6.1.2) (NPR-A) (Atrial natriuretic peptide A-type receptor) - Rattus norvegicus (Rat)

Match to Query 208: 2125.805448 from(1063.910000,2+)
Title: File: Qtrap0014366.wiff, Sample: JS TiO2 2 (sample number 1), Elution: 61.177 min, Period: 1, Cycle(s): 1109 (Experiment 3) (Charge not auto determined)
Data file C:\Dokumente und Einstellungen\Juliane\Eigene Dateien\Qtrap-files\2008\20032008\Qtrap0014366-1.mgf

Click mouse within plot area to zoom in by factor of two about that point
Or, Plot from 100 to 1700 Da Full range

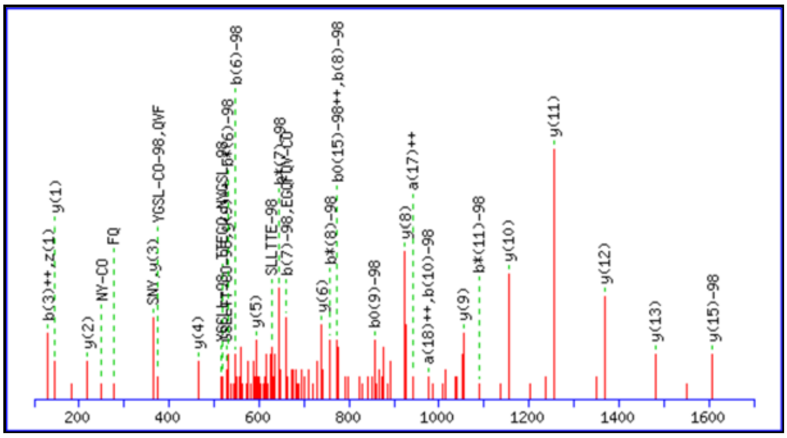

Monoisotopic mass of neutral peptide Mr(calc): 2125.9722
Fixed modifications: Carbamidomethyl (C)
Variable modifications:
S6 : Phospho (ST), with neutral losses 97.9769(shown in table), 0.0000
Ions Score: 100 **Expect:** 5.2e-009
Matches (Bold Red): 41/542 fragment ions using 47 most intense peaks

#	Immon.	a	a^{++}	b	b^{++}	b*	b^{*++}	b^0	b^{0++}	Seq.	y	y^{++}	z	z^{++}	#
1	30.0338	30.0338	15.5206	58.0287	29.5180					G					19
2	60.0444	117.0659	59.0366	145.0608	73.0340			127.0502	64.0287	S	1971.9811	986.4942	1954.9545	977.9809	18
3	87.0553	231.1088	116.0580	259.1037	**130.0555**	242.0771	121.5422	241.0931	121.0502	N	1884.9490	942.9782	1867.9225	934.4649	17
4	136.0757	394.1721	197.5897	422.1670	211.5872	405.1405	203.0739	404.1565	202.5819	Y	1770.9061	885.9567	1753.8796	877.4434	16
5	30.0338	451.1936	226.1004	479.1885	240.0979	462.1619	231.5846	461.1779	231.0926	G	**1607.8428**	804.4250	1590.8162	795.9118	15
6	42.0338	520.2150	260.6112	**548.2099**	274.6086	**531.1834**	266.0953	530.1994	265.6033	S	1550.8213	775.9143	1533.7948	767.4010	14
7	86.0964	633.2991	317.1532	**661.2940**	331.1506	**644.2675**	322.6374	643.2834	322.1454	L	**1481.7999**	741.4036	1464.7733	732.8903	13
8	86.0964	746.3832	373.6952	**774.3781**	387.6927	**757.3515**	379.1794	756.3675	378.6874	L	**1368.7158**	684.8615	1351.6892	676.3483	12
9	74.0600	847.4308	424.2191	875.4258	438.2165	858.3992	429.7032	**857.4152**	429.2112	T	**1255.6317**	628.3195	1238.6052	619.8062	11
10	74.0600	948.4785	474.7429	**976.4734**	488.7404	959.4469	480.2271	958.4629	479.7351	T	**1154.5841**	577.7957	1137.5575	569.2824	10
11	102.0550	1077.5211	539.2642	1105.5160	553.2616	**1088.4895**	544.7484	1087.5055	544.2564	E	**1053.5364**	**527.2718**	1036.5098	518.7586	9
12	30.0338	1134.5426	567.7749	1162.5375	581.7724	1145.5109	573.2591	1144.5269	572.7671	G	**924.4938**	462.7505	907.4672	454.2373	8
13	101.0709	1262.6012	631.8042	1290.5961	645.8017	1273.5695	637.2884	1272.5855	636.7964	Q	867.4723	434.2398	850.4458	425.7265	7
14	120.0808	1409.6696	705.3384	1437.6645	719.3359	1420.6379	710.8226	1419.6539	710.3306	F	**739.4137**	370.2105	722.3872	361.6972	6
15	101.0709	1537.7281	769.3677	1565.7231	783.3652	1548.6965	774.8519	1547.7125	**774.3599**	Q	**592.3453**	296.6763	575.3188	288.1630	5
16	72.0808	1636.7966	818.9019	1664.7915	832.8994	1647.7649	824.3861	1646.7809	823.8941	V	**464.2867**	232.6470	447.2602	224.1337	4
17	120.0808	1783.8650	892.4361	1811.8599	906.4336	1794.8333	897.9203	1793.8493	897.4283	F	**365.2183**	183.1128	348.1918	174.5995	3
18	44.0495	1854.9021	927.9547	1882.8970	941.9521	1865.8705	933.4389	1864.8864	932.9469	A	**218.1499**	109.5786	201.1234	101.0653	2
19	101.1073									K	**147.1128**	74.0600	**130.0863**	65.5468	1

Seq	ya	yb	Seq	ya	yb	Seq	ya	yb
SN	174.0873	202.0822	SNY	337.1506	**365.1456**	SNYG	394.1721	422.1670
SNYGS	463.1936	491.1885	SNYGSL	576.2776	604.2725	SNYGSLL	689.3617	717.3566
NY	**250.1186**	278.1135	NYG	307.1401	335.1350	NYGS	376.1615	404.1565
NYGSL	489.2456	**517.2405**	NYGSLL	602.3297	630.3246	YG	193.0972	221.0921
YGS	262.1186	290.1135	YGSL	**375.2027**	403.1976	YGSLL	488.2867	**516.2817**
YGSLLT	589.3344	617.3293	YGSLLTT	690.3821	718.3770	GS	99.0553	127.0502
GSL	212.1393	240.1343	GSLL	325.2234	353.2183	GSLLT	426.2711	454.2660

**GSLLTT**	**527.3188**	555.3137	**GSLLTTE**	656.3614	684.3563	**SL**	155.1179	183.1128
**SLL**	268.2019	296.1969	**SLLT**	369.2496	397.2445	**SLLTT**	470.2973	498.2922
**SLLTTE**	599.3399	**627.3348**	**SLLTTEG**	656.3614	684.3563	**LL**	199.1805	227.1754
**LLT**	300.2282	328.2231	**LLTT**	401.2758	429.2708	**LLTTE**	530.3184	558.3134
**LLTTEG**	587.3399	615.3348	**LT**	187.1441	215.1390	**LTT**	288.1918	316.1867
**LTTE**	417.2344	445.2293	**LTTEG**	474.2558	502.2508	**LTTEGQ**	602.3144	630.3093
**TT**	175.1077	203.1026	**TTE**	304.1503	332.1452	**TTEG**	361.1718	389.1667
**TTEGQ**	489.2304	**517.2253**	**TTEGQF**	636.2988	664.2937	**TE**	203.1026	231.0975
**TEG**	260.1241	288.1190	**TEGQ**	388.1827	416.1776	**TEGQF**	535.2511	563.2460
**TEGQFQ**	663.3097	691.3046	**EG**	159.0764	187.0713	**EGQ**	287.1350	315.1299
**EGQF**	434.2034	462.1983	**EGQFQ**	562.2620	590.2569	**EGQFQV**	**661.3304**	689.3253
**GQ**	158.0924	186.0873	**GQF**	305.1608	333.1557	**GQFQ**	433.2194	461.2143
**GQFQV**	532.2878	560.2827	**GQFQVF**	679.3562	707.3511	**QF**	248.1394	**276.1343**
**QFQ**	376.1979	404.1928	**QFQV**	475.2663	503.2613	**QFQVF**	622.3348	650.3297
**QFQVFA**	693.3719	721.3668	**FQ**	248.1394	**276.1343**	**FQV**	347.2078	**375.2027**
**FQVF**	494.2762	522.2711	**FQVFA**	565.3133	593.3082	**QV**	200.1394	228.1343
**QVF**	347.2078	**375.2027**	**QVFA**	418.2449	446.2398	**VF**	219.1492	247.1441
**VFA**	290.1863	318.1812	**FA**	191.1179	219.1128			

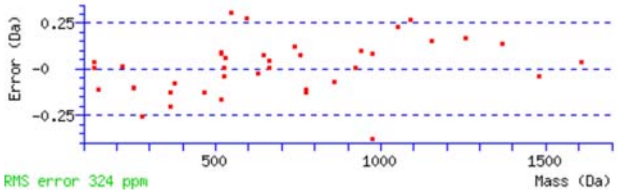

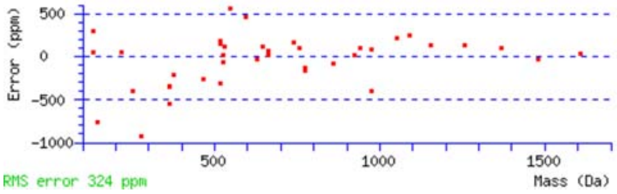

NCBI **BLAST** search of GSNYGSLLTTEGQFQVFAK
(Parameters: blastp, nr protein database, expect=20000, no filter, PAM30)
Other BLAST web gateways

All matches to this query

Score	Mr(calc):	Delta	Sequence
100.1	2125.9722	-0.1667	GSNYGSLLTTEGQFQVFAK
84.0	2125.9722	-0.1667	GSNYGSLLTTEGQFQVFAK
70.8	2125.9722	-0.1667	GSNYGSLLTTEGQFQVFAK
68.4	2125.9722	-0.1667	GSNYGSLLTTEGQFQVFAK
62.6	2125.9722	-0.1667	GSNYGSLLTTEGQFQVFAK
16.0	2126.0565	-0.2511	EHMLVLGEESQLTLGETPK
9.9	2125.8906	-0.0852	YGVYEAIFTMLSSLMNK
9.9	2125.8906	-0.0852	YGVYEAIFTMLSSLMNK
8.1	2125.8906	-0.0852	YGVYEAIFTMLSSLMNK
8.1	2125.8906	-0.0852	YGVYEAIFTMLSSLMNK

Mascot: http://www.matrixscience.com/

Mascot Search Results

Peptide View

MS/MS Fragmentation of **GSNYGSLLTTEGQFQVFAK**
Found in **ANPRA_RAT**, Atrial natriuretic peptide receptor A precursor (ANP-A) (ANPRA) (GC-A) (Guanylate cyclase) (EC 4.6.1.2) (NPR-A) (Atrial natriuretic peptide A-type receptor) - Rattus norvegicus (Rat)

Match to Query 358: 2126.197839 from(709.739889,3+)
Title: File: QstarE03478.wiff, Sample: JS TiO2 Probe b (sample number 1), Elution: 128.266 to 128.327 min, Period: 1, Cycle(s): 12053-12055 (Experiment 2)
Data file C:\Dokumente und Einstellungen\Juliane\Eigene Dateien\QStar-Files\18042008\QstarE03478.mgf

Click mouse within plot area to zoom in by factor of two about that point
Or, Plot from 100 to 1400 Da Full range

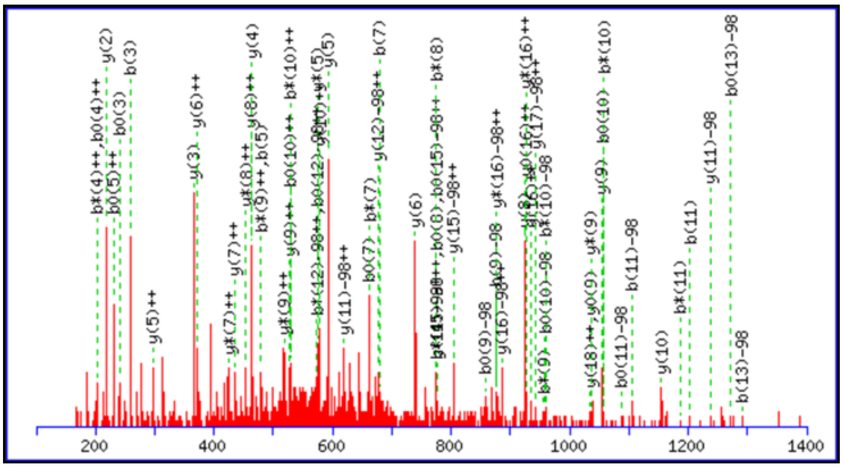

Monoisotopic mass of neutral peptide Mr(calc): 2125.9722
Fixed modifications: Carbamidomethyl (C)
Variable modifications:
T9 : Phospho (ST), with neutral losses 97.9769(shown in table), 0.0000
Ions Score: 57 **Expect:** 6.5e-005
Matches (Bold Red): 63/302 fragment ions using 99 most intense peaks

#	b	b^{++}	b*	b^{*++}	b^0	b^{0++}	Seq.	y	y^{++}	y*	y^{*++}	y^0	y^{0++}	#
1	58.0287	29.5180					G							19
2	145.0608	73.0340			127.0502	64.0287	S	1971.9811	986.4942	1954.9545	977.9809	1953.9705	977.4889	18
3	**259.1037**	130.0555	242.0771	121.5422	**241.0931**	121.0502	N	1884.9490	**942.9782**	1867.9225	934.4649	1866.9385	933.9729	17
4	422.1670	211.5872	405.1405	**203.0739**	404.1565	**202.5819**	Y	1770.9061	**885.9567**	1753.8796	**877.4434**	1752.8955	876.9514	16
5	**479.1885**	240.0979	462.1619	231.5846	461.1779	**231.0926**	G	1607.8428	**804.4250**	1590.8162	795.9118	1589.8322	795.4197	15
6	566.2205	283.6139	549.1940	275.1006	548.2100	274.6086	S	1550.8213	**775.9143**	1533.7948	767.4010	1532.8108	766.9090	14
7	**679.3046**	340.1559	**662.2780**	331.6427	**661.2940**	331.1506	L	1463.7893	732.3983	1446.7627	723.8850	1445.7787	723.3930	13
8	792.3886	396.6980	**775.3621**	388.1847	**774.3781**	387.6927	L	1350.7052	**675.8562**	1333.6787	667.3430	1332.6947	666.8510	12
9	**875.4258**	438.2165	858.3992	429.7032	**857.4152**	429.2112	T	**1237.6212**	**619.3142**	1220.5946	610.8009	1219.6106	610.3089	11
10	976.4734	488.7404	**959.4469**	480.2271	**958.4629**	479.7351	T	**1154.5841**	**577.7957**	1137.5575	569.2824	1136.5735	568.7904	10
11	**1105.5160**	553.2616	1088.4895	544.7484	**1087.5055**	544.2564	E	**1053.5364**	**527.2718**	**1036.5098**	**518.7586**	**1035.5258**	518.2665	9
12	1162.5375	581.7724	1145.5109	**573.2591**	1144.5269	**572.7671**	G	**924.4938**	**462.7505**	907.4672	**454.2373**			8
13	**1290.5961**	645.8017	1273.5695	637.2884	**1272.5855**	636.7964	Q	867.4723	**434.2398**	850.4458	**425.7265**			7
14	1437.6645	719.3359	1420.6379	710.8226	1419.6539	710.3306	F	**739.4137**	**370.2105**	722.3872	361.6972			6
15	1565.7231	783.3652	1548.6965	**774.8519**	1547.7125	**774.3599**	Q	**592.3453**	**296.6763**	**575.3188**	288.1630			5
16	1664.7915	832.8994	1647.7649	824.3861	1646.7809	823.8941	V	**464.2867**	232.6470	447.2602	224.1337			4
17	1811.8599	906.4336	1794.8333	897.9203	1793.8493	897.4283	F	**365.2183**	183.1128	348.1918	174.5995			3
18	1882.8970	941.9521	1865.8705	933.4389	1864.8864	932.9469	A	**218.1499**	109.5786	201.1234	101.0653			2
19							K	147.1128	74.0600	130.0863	65.5468			1

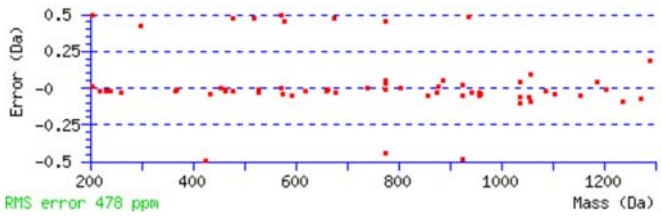

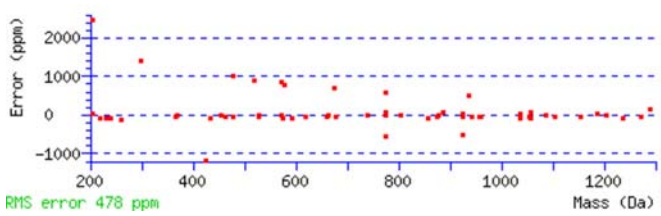

NCBI **BLAST** search of GSNYGSLLTTEGQFQVFAK
(Parameters: blastp, nr protein database, expect=20000, no filter, PAM30)
Other BLAST web gateways

All matches to this query

Score	Mr(calc):	Delta	Sequence
57.0	2125.9722	0.2257	GSNYGSLLTTEGQFQVFAK
54.9	2125.9722	0.2257	GSNYGSLLTTEGQFQVFAK
54.5	2125.9722	0.2257	GSNYGSLLTTEGQFQVFAK
54.5	2125.9722	0.2257	GSNYGSLLTTEGQFQVFAK
45.1	2125.9722	0.2257	GSNYGSLLTTEGQFQVFAK
3.8	2125.9924	0.2054	GLMTLQALYGTIPQIFGK
3.3	2125.9771	0.2207	VYENVTGLVKAVIEMSSK
2.0	2125.9771	0.2207	VYENVTGLVKAVIEMSSK
1.9	2125.9205	0.2774	HLPGPGGNDEPTDLEELEK

Mascot: http://www.matrixscience.com/

Mascot Search Results

Peptide View

MS/MS Fragmentation of **GSNYGSLLTTEGQFQVFAK**
Found in **ANPRA_RAT**, Atrial natriuretic peptide receptor A precursor (ANP-A) (ANPRA) (GC-A) (Guanylate cyclase) (EC 4.6.1.2) (NPR-A) (Atrial natriuretic peptide A-type receptor) - Rattus norvegicus (Rat)

Match to Query 161: 2205.825448 from(1103.920000,2+)
Title: File: Qtrap0014365.wiff, Sample: JS TiO2 1 (sample number 1), Elution: 71.061 to 71.174 min, Period: 1, Cycle(s): 1586-1587 (Experiment 3) (Charge not auto determined)
Data file C:\Dokumente und Einstellungen\Juliane\Eigene Dateien\Qtrap-files\2008\20032008\Qtrap0014365-1.mgf

Click mouse within plot area to zoom in by factor of two about that point
Or, Plot from 100 to 1700 Da Full range

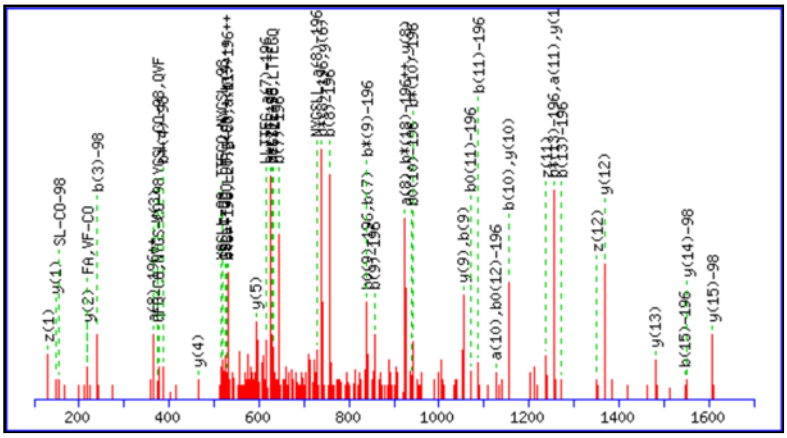

Monoisotopic mass of neutral peptide Mr(calc): 2205.9385
Fixed modifications: Carbamidomethyl (C)
Variable modifications:
S2 : Phospho (ST), with neutral losses 97.9769(shown in table), 0.0000
S6 : Phospho (ST), with neutral losses 97.9769(shown in table), 0.0000
Ions Score: 78 **Expect:** 8.6e-007
Matches (Bold Red): 69/581 fragment ions using 95 most intense peaks

#	Immon.	a	a^{++}	b	b^{++}	b*	b^{*++}	b^0	b^{0++}	Seq.	y	y^{++}	z	z^{++}	#
1	30.0338	30.0338	15.5206	58.0287	29.5180					G					19
2	42.0338	99.0553	50.0313	127.0502	64.0287			109.0396	55.0235	S	1953.9705	977.4889	1936.9439	968.9756	18
3	87.0553	213.0982	107.0527	**241.0931**	121.0502	224.0666	112.5369	223.0826	112.0449	N	1884.9490	942.9782	1867.9225	934.4649	17
4	136.0757	**376.1615**	188.5844	404.1565	202.5819	**387.1299**	194.0686	386.1459	193.5766	Y	1770.9061	885.9567	1753.8796	877.4434	16
5	30.0338	433.1830	217.0951	461.1779	231.0926	444.1514	222.5793	443.1674	222.0873	G	**1607.8428**	804.4250	1590.8162	795.9118	15
6	42.0338	502.2045	251.6059	**530.1994**	265.6033	513.1728	257.0900	512.1888	256.5980	S	**1550.8213**	775.9143	1533.7948	767.4010	14
7	86.0964	**615.2885**	308.1479	**643.2834**	322.1454	**626.2569**	313.6321	625.2729	313.1401	L	**1481.7999**	741.4036	1464.7733	732.8903	13
8	86.0964	**728.3726**	**364.6899**	**756.3675**	378.6874	**739.3410**	370.1741	738.3569	369.6821	L	**1368.7158**	684.8615	**1351.6892**	676.3483	12
9	74.0600	829.4203	415.2138	**857.4152**	429.2112	**840.3886**	420.6980	**839.4046**	420.2059	T	**1255.6317**	628.3195	**1238.6052**	619.8062	11
10	74.0600	930.4679	465.7376	958.4629	479.7351	**941.4363**	471.2218	**940.4523**	470.7298	T	**1154.5841**	577.7957	1137.5575	569.2824	10
11	102.0550	1059.5105	**530.2589**	**1087.5055**	544.2564	1070.4789	535.7431	**1069.4949**	535.2511	E	**1053.5364**	**527.2718**	1036.5098	518.7586	9
12	30.0338	1116.5320	558.7696	1144.5269	572.7671	1127.5004	564.2538	**1126.5163**	563.7618	G	**924.4938**	462.7505	907.4672	454.2373	8
13	101.0709	1244.5906	622.7989	**1272.5855**	636.7964	**1255.5589**	628.2831	1254.5749	627.7911	Q	867.4723	434.2398	850.4458	425.7265	7
14	120.0808	1391.6590	696.3331	1419.6539	710.3306	1402.6274	701.8173	1401.6433	701.3253	F	**739.4137**	370.2105	722.3872	361.6972	6
15	101.0709	1519.7176	760.3624	**1547.7125**	774.3599	1530.6859	765.8466	1529.7019	765.3546	Q	**592.3453**	296.6763	575.3188	288.1630	5
16	72.0808	1618.7860	809.8966	1646.7809	823.8941	1629.7544	815.3808	1628.7703	814.8888	V	**464.2867**	232.6470	447.2602	224.1337	4
17	120.0808	1765.8544	883.4308	1793.8493	897.4283	1776.8228	888.9150	1775.8387	888.4230	F	**365.2183**	183.1128	348.1918	174.5995	3
18	44.0495	1836.8915	918.9494	1864.8864	932.9469	1847.8599	**924.4336**	1846.8759	923.9416	A	**218.1499**	109.5786	201.1234	101.0653	2
19	101.1073									K	**147.1128**	74.0600	**130.0863**	65.5468	1

Seq	ya	yb	Seq	ya	yb	Seq	ya	yb
SN	156.0767	184.0717	SNY	319.1401	347.1350	SNYG	**376.1615**	404.1565
SNYGS	445.1830	473.1779	SNYGSL	558.2671	586.2620	SNYGSLL	671.3511	699.3460
NY	250.1186	278.1135	NYG	307.1401	335.1350	NYGS	**376.1615**	404.1565
NYGSL	489.2456	**517.2405**	NYGSLL	602.3297	**630.3246**	YG	193.0972	221.0921
YGS	262.1186	290.1135	YGSL	**375.2027**	403.1976	YGSLL	488.2867	**516.2817**
YGSLLT	589.3344	617.3293	YGSLLTT	690.3821	718.3770	GS	99.0553	127.0502

**GSL**	212.1393	240.1343	**GSLL**	325.2234	353.2183	**GSLLT**	426.2711	454.2660
**GSLLTT**	**527.3188**	555.3137	**GSLLTTE**	656.3614	684.3563	**SL**	**155.1179**	183.1128
**SLL**	268.2019	296.1969	**SLLT**	369.2496	397.2445	**SLLTT**	470.2973	498.2922
**SLLTTE**	599.3399	**627.3348**	**SLLTTEG**	656.3614	684.3563	**LL**	199.1805	227.1754
**LLT**	300.2282	328.2231	**LLTT**	401.2758	429.2708	**LLTTE**	**530.3184**	558.3134
**LLTTEG**	587.3399	**615.3348**	**LT**	187.1441	215.1390	**LTT**	288.1918	316.1867
**LTTE**	417.2344	445.2293	**LTTEG**	474.2558	502.2508	**LTTEGQ**	602.3144	**630.3093**
**TT**	175.1077	203.1026	**TTE**	304.1503	332.1452	**TTEG**	361.1718	389.1667
**TTEGQ**	489.2304	**517.2253**	**TTEGQF**	636.2988	664.2937	**TE**	203.1026	231.0975
**TEG**	260.1241	288.1190	**TEGQ**	388.1827	416.1776	**TEGQF**	535.2511	563.2460
**TEGQFQ**	663.3097	691.3046	**EG**	159.0764	187.0713	**EGQ**	287.1350	315.1299
**EGQF**	434.2034	462.1983	**EGQFQ**	562.2620	590.2569	**EGQFQV**	661.3304	689.3253
**GQ**	158.0924	186.0873	**GQF**	305.1608	333.1557	**GQFQ**	433.2194	461.2143
**GQFQV**	532.2878	560.2827	**GQFQVF**	679.3562	707.3511	**QF**	248.1394	276.1343
**QFQ**	**376.1979**	404.1928	**QFQV**	475.2663	503.2613	**QFQVF**	622.3348	650.3297
**QFQVFA**	693.3719	721.3668	**FQ**	248.1394	276.1343	**FQV**	347.2078	**375.2027**
**FQVF**	494.2762	522.2711	**FQVFA**	565.3133	593.3082	**QV**	200.1394	228.1343
**QVF**	347.2078	**375.2027**	**QVFA**	418.2449	446.2398	**VF**	**219.1492**	247.1441
**VFA**	290.1863	318.1812	**FA**	191.1179	**219.1128**			

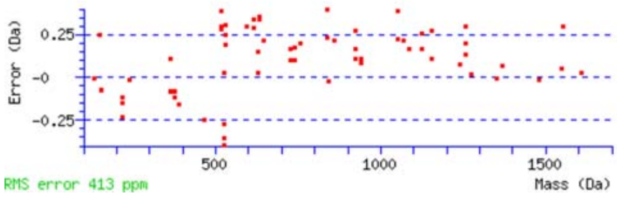

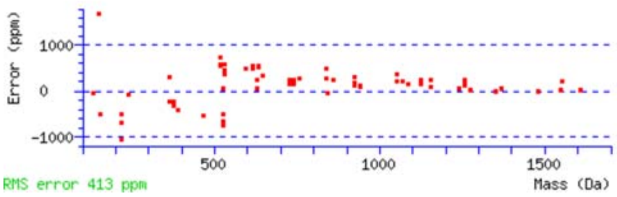

NCBI **BLAST** search of GSNYGSLLTTEGQFQVFAK
(Parameters: blastp, nr protein database, expect=20000, no filter, PAM30)
Other BLAST web gateways

All matches to this query

Score	Mr(calc):	Delta	Sequence
78.2	2205.9385	-0.1130	GSNYGSLLTTEGQFQVFAK
71.8	2205.9385	-0.1130	GSNYGSLLTTEGQFQVFAK
64.5	2205.9385	-0.1130	GSNYGSLLTTEGQFQVFAK
62.3	2205.9385	-0.1130	GSNYGSLLTTEGQFQVFAK
57.3	2205.9385	-0.1130	GSNYGSLLTTEGQFQVFAK
55.8	2205.9385	-0.1130	GSNYGSLLTTEGQFQVFAK
50.1	2205.9385	-0.1130	GSNYGSLLTTEGQFQVFAK
47.8	2205.9385	-0.1130	GSNYGSLLTTEGQFQVFAK
40.3	2205.9385	-0.1130	GSNYGSLLTTEGQFQVFAK
13.0	2205.9385	-0.1130	GSNYGSLLTTEGQFQVFAK

Mascot: http://www.matrixscience.com/

Mascot Search Results

Peptide View

MS/MS Fragmentation of **GSNYGSLLTTEGQFQVFAK**
Found in **ANPRA_RAT**, Atrial natriuretic peptide receptor A precursor (ANP-A) (ANPRA) (GC-A) (Guanylate cyclase) (EC 4.6.1.2) (NPR-A) (Atrial natriuretic peptide A-type receptor) - Rattus norvegicus (Rat)

Match to Query 162: 2205.848172 from(736.290000,3+)
Title: File: Qtrap0014365.wiff, Sample: JS TiO2 1 (sample number 1), Elution: 69.124 to 69.237 min, Period: 1, Cycle(s): 1536-1537 (Experiment 3) (Charge not auto determined)
Data file C:\Dokumente und Einstellungen\Juliane\Eigene Dateien\Qtrap-files\2008\20032008\Qtrap0014365-1.mgf

Click mouse within plot area to zoom in by factor of two about that point
Or, Plot from 100 to 1300 Da Full range

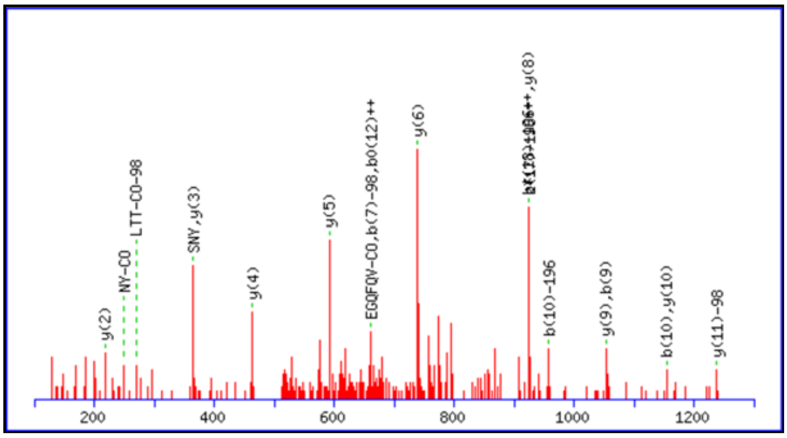

Monoisotopic mass of neutral peptide Mr(calc): 2205.9385
Fixed modifications: Carbamidomethyl (C)
Variable modifications:
S6 : Phospho (ST), with neutral losses 97.9769(shown in table), 0.0000
T9 : Phospho (ST), with neutral losses 97.9769(shown in table), 0.0000
Ions Score: 87 **Expect:** 1.3e-007
Matches (Bold Red): 20/589 fragment ions using 15 most intense peaks

#	Immon.	a	a^{++}	b	b^{++}	b*	b^{*++}	b^0	b^{0++}	Seq.	y	y^{++}	z	z^{++}	#
1	30.0338	30.0338	15.5206	58.0287	29.5180					G					19
2	60.0444	117.0659	59.0366	145.0608	73.0340			127.0502	64.0287	S	1953.9705	977.4889	1936.9439	968.9756	18
3	87.0553	231.1088	116.0580	259.1037	130.0555	242.0771	121.5422	241.0931	121.0502	N	1866.9385	933.9729	1849.9119	**925.4596**	17
4	136.0757	394.1721	197.5897	422.1670	211.5872	405.1405	203.0739	404.1565	202.5819	Y	1752.8955	876.9514	1735.8690	868.4381	16
5	30.0338	451.1936	226.1004	479.1885	240.0979	462.1619	231.5846	461.1779	231.0926	G	1589.8322	795.4197	1572.8057	786.9065	15
6	42.0338	520.2150	260.6112	548.2099	274.6086	531.1834	266.0953	530.1994	265.6033	S	1532.8107	766.9090	1515.7842	758.3957	14
7	86.0964	633.2991	317.1532	**661.2940**	331.1506	644.2675	322.6374	643.2834	322.1454	L	1463.7893	732.3983	1446.7627	723.8850	13
8	86.0964	746.3832	373.6952	774.3781	387.6927	757.3515	379.1794	756.3675	378.6874	L	1350.7052	675.8562	1333.6787	667.3430	12
9	56.0495	829.4203	415.2138	857.4152	429.2112	840.3886	420.6980	839.4046	420.2059	T	**1237.6212**	619.3142	1220.5946	610.8009	11
10	74.0600	930.4679	465.7376	**958.4629**	479.7351	941.4363	471.2218	940.4523	470.7298	T	**1154.5841**	577.7957	1137.5575	569.2824	10
11	102.0550	1059.5105	530.2589	1087.5055	544.2564	1070.4789	535.7431	1069.4949	535.2511	E	**1053.5364**	527.2718	1036.5098	518.7586	9
12	30.0338	1116.5320	558.7696	1144.5269	572.7671	1127.5004	564.2538	1126.5163	563.7618	G	**924.4938**	462.7505	907.4672	454.2373	8
13	101.0709	1244.5906	622.7989	1272.5855	636.7964	1255.5589	628.2831	1254.5749	627.7911	Q	867.4723	434.2398	850.4458	425.7265	7
14	120.0808	1391.6590	696.3331	1419.6539	710.3306	1402.6274	701.8173	1401.6433	701.3253	F	**739.4137**	370.2105	722.3872	361.6972	6
15	101.0709	1519.7176	760.3624	1547.7125	774.3599	1530.6859	765.8466	1529.7019	765.3546	Q	**592.3453**	296.6763	575.3188	288.1630	5
16	72.0808	1618.7860	809.8966	1646.7809	823.8941	1629.7544	815.3808	1628.7703	814.8888	V	**464.2867**	232.6470	447.2602	224.1337	4
17	120.0808	1765.8544	883.4308	1793.8493	897.4283	1776.8228	888.9150	1775.8387	888.4230	F	**365.2183**	183.1128	348.1918	174.5995	3
18	44.0495	1836.8915	918.9494	1864.8864	932.9469	1847.8599	**924.4336**	1846.8759	923.9416	A	**218.1499**	109.5786	201.1234	101.0653	2
19	101.1073									K	147.1128	74.0600	130.0863	65.5468	1

Seq	ya	yb	Seq	ya	yb	Seq	ya	yb
SN	174.0873	202.0822	SNY	337.1506	**365.1456**	SNYG	394.1721	422.1670
SNYGS	463.1936	491.1885	SNYGSL	576.2776	604.2725	SNYGSLL	689.3617	717.3566
NY	**250.1186**	278.1135	NYG	307.1401	335.1350	NYGS	376.1615	404.1565
NYGSL	489.2456	517.2405	NYGSLL	602.3297	630.3246	NYGSLLT	685.3668	713.3617
YG	193.0972	221.0921	YGS	262.1186	290.1135	YGSL	375.2027	403.1976
YGSLL	488.2867	516.2817	YGSLLT	571.3238	599.3188	YGSLLTT	672.3715	700.3664

**GS**	99.0553	127.0502	**GSL**	212.1393	240.1343	**GSLL**	325.2234	353.2183
**GSLLT**	408.2605	436.2554	**GSLLTT**	509.3082	537.3031	**GSLLTTE**	638.3508	666.3457
**GSLLTTEG**	695.3723	723.3672	**SL**	155.1179	183.1128	**SLL**	268.2019	296.1969
**SLLT**	351.2391	379.2340	**SLLTT**	452.2867	480.2816	**SLLTTE**	581.3293	609.3242
**SLLTTEG**	638.3508	666.3457	**LL**	199.1805	227.1754	**LLT**	282.2176	310.2125
**LLTT**	383.2653	411.2602	**LLTTE**	512.3079	540.3028	**LLTTEG**	569.3293	597.3242
**LLTTEGQ**	697.3879	725.3828	**LT**	169.1335	197.1284	**LTT**	**270.1812**	298.1761
**LTTE**	399.2238	427.2187	**LTTEG**	456.2453	484.2402	**LTTEGQ**	584.3038	612.2988
**TT**	157.0971	185.0921	**TTE**	286.1397	314.1347	**TTEG**	343.1612	371.1561
**TTEGQ**	471.2198	499.2147	**TTEGQF**	618.2882	646.2831	**TE**	203.1026	231.0975
**TEG**	260.1241	288.1190	**TEGQ**	388.1827	416.1776	**TEGQF**	535.2511	563.2460
**TEGQFQ**	663.3097	691.3046	**EG**	159.0764	187.0713	**EGQ**	287.1350	315.1299
**EGQF**	434.2034	462.1983	**EGQFQ**	562.2620	590.2569	**EGQFQV**	**661.3304**	689.3253
**GQ**	158.0924	186.0873	**GQF**	305.1608	333.1557	**GQFQ**	433.2194	461.2143
**GQFQV**	532.2878	560.2827	**GQFQVF**	679.3562	707.3511	**QF**	248.1394	276.1343
**QFQ**	376.1979	404.1928	**QFQV**	475.2663	503.2613	**QFQVF**	622.3348	650.3297
**QFQVFA**	693.3719	721.3668	**FQ**	248.1394	276.1343	**FQV**	347.2078	375.2027
**FQVF**	494.2762	522.2711	**FQVFA**	565.3133	593.3082	**QV**	200.1394	228.1343
**QVF**	347.2078	375.2027	**QVFA**	418.2449	446.2398	**VF**	219.1492	247.1441
**VFA**	290.1863	318.1812	**FA**	191.1179	219.1128			

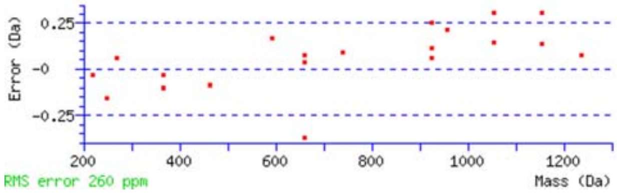

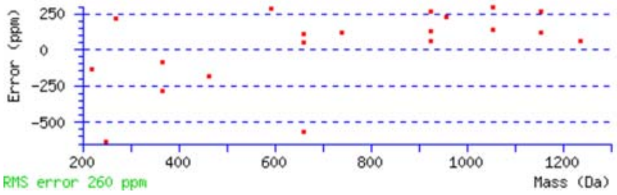

NCBI **BLAST** search of GSNYGSLLTTEGQFQVFAK
(Parameters: blastp, nr protein database, expect=20000, no filter, PAM30)
Other BLAST web gateways

All matches to this query

Score	Mr(calc):	Delta	Sequence
86.6	2205.9385	-0.0903	GSNYGSLLTTEGQFQVFAK
86.6	2205.9385	-0.0903	GSNYGSLLTTEGQFQVFAK
74.4	2205.9385	-0.0903	GSNYGSLLTTEGQFQVFAK
74.3	2205.9385	-0.0903	GSNYGSLLTTEGQFQVFAK
74.3	2205.9385	-0.0903	GSNYGSLLTTEGQFQVFAK
63.3	2205.9385	-0.0903	GSNYGSLLTTEGQFQVFAK
58.7	2205.9385	-0.0903	GSNYGSLLTTEGQFQVFAK
58.6	2205.9385	-0.0903	GSNYGSLLTTEGQFQVFAK
58.6	2205.9385	-0.0903	GSNYGSLLTTEGQFQVFAK
48.5	2205.9385	-0.0903	GSNYGSLLTTEGQFQVFAK

Mascot: http://www.matrixscience.com/
