# Supplementary material for: Homologous desensitization of guanylyl cyclase A, the receptor for atrial natriuretic peptide, is associated with a complex phosphorylation pattern
Source: FEBS J. 2010 Jun;277(11):2440–53. doi: 10.1111/j.1742-4658.2010.07658.x (PMC2901513; doi:10.1111/j.1742-4658.2010.07658.x)
Supplement: Supplementary file 3 [file ejb0277-2440-SD3.pdf]

Supplementary Fig. S3. Fragment ion spectra of all identified tryptic GC-A phosphopeptides obtained from GC-A, endogenously expressed in murine microvascular myocardial endothelial cells**.** Note that ion masses within the included tables can refer to intact b- and y-ions and to b- and y-ions after ß-elimination of phosphoric acid. *, neutral loss of NH_3; ^{0}, neutral loss of H_2O.

Mascot Search Results

Peptide View

MS/MS Fragmentation of **WEDLQPSSLER**
Found in **IPI00111183**, Tax_Id=10090 Gene_Symbol=Npr1 Atrial natriuretic peptide receptor A

Match to Query 729: 1438.613724 from(720.314138,2+)
Title: orbitrap002312.1333.1333.2.dta
Data file C:\Dokumente und Einstellungen\Juliane\Eigene Dateien\Orbitrap-Files\orbitrap002312.RAW.mgf

Click mouse within plot area to zoom in by factor of two about that point
Or, Plot from 100 to 1400 Da Full range

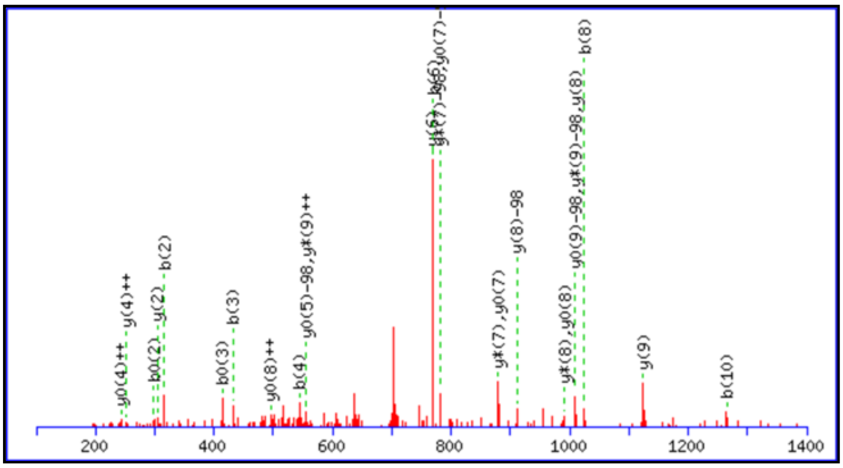

Monoisotopic mass of neutral peptide Mr(calc): 1438.6129
Fixed modifications: Carbamidomethyl (C)
Variable modifications:
S7 : Phospho (ST), with neutral losses 0.0000(shown in table), 97.9769
Ions Score: 31 **Expect:** 0.024
Matches (Bold Red): 26/168 fragment ions using 43 most intense peaks

#	b	b^{++}	b*	b^{*++}	b^0	b^{0++}	Seq.	y	y^{++}	y*	y^{*++}	y^0	y^{0++}	#
1	187.0866	94.0469					W							11
2	**316.1292**	158.5682			**298.1186**	149.5629	E	1253.5409	627.2741	1236.5144	618.7608	1235.5304	618.2688	10
3	**431.1561**	216.0817			**413.1456**	207.0764	D	**1124.4983**	562.7528	1107.4718	**554.2395**	1106.4878	553.7475	9
4	**544.2402**	272.6237			526.2296	263.6185	L	**1009.4714**	505.2393	**992.4448**	496.7261	**991.4608**	**496.2341**	8
5	672.2988	336.6530	655.2722	328.1397	654.2882	327.6477	Q	896.3873	448.6973	**879.3608**	440.1840	**878.3768**	439.6920	7
6	**769.3515**	385.1794	752.3250	376.6661	751.3410	376.1741	P	**768.3288**	384.6680	751.3022	376.1547	750.3182	375.6627	6
7	936.3499	468.6786	919.3233	460.1653	918.3393	459.6733	S	671.2760	336.1416	654.2494	327.6284	653.2654	327.1363	5
8	**1023.3819**	512.1946	1006.3554	503.6813	1005.3713	503.1893	S	504.2776	**252.6425**	487.2511	244.1292	486.2671	**243.6372**	4
9	1136.4660	568.7366	1119.4394	560.2234	1118.4554	559.7313	L	417.2456	209.1264	400.2191	200.6132	399.2350	200.1212	3
10	**1265.5086**	633.2579	1248.4820	624.7446	1247.4980	624.2526	E	**304.1615**	152.5844	287.1350	144.0711	286.1510	143.5791	2
11							R	175.1190	88.0631	158.0924	79.5498			1

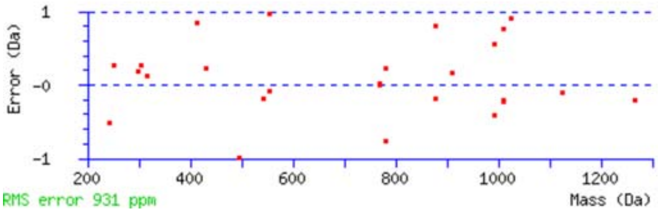

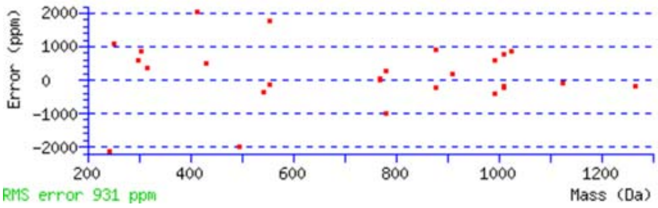

NCBI **BLAST** search of WEDLQPSSLER
(Parameters: blastp, nr protein database, expect=20000, no filter, PAM30)
Other BLAST web gateways

All matches to this query

Score	Mr(calc):	Delta	Sequence
31.2	1438.6129	0.0008	WEDLQPSSLER

30.0	1438.6129	0.0008	WEDLQPSSLER
4.7	1437.6166	0.9971	NENYKSLVRR
4.2	1438.6082	0.0056	LPCHENQECPR
3.6	1437.6095	1.0043	VATPPPSAQPWK
3.0	1438.6129	0.0008	EGEPWSGAAAAVSK
2.6	1437.6072	1.0066	SHVDSKCEQIR
1.8	1438.6159	-0.0022	TAYKFAKSHAR
0.5	1438.6129	0.0008	GLYKESGSAYER
0.0	1438.6089	0.0048	SDISQPASQDGVR

Mascot: http://www.matrixscience.com/

Mascot Search Results

Peptide View

MS/MS Fragmentation of **SAGSRLTLSGR**
Found in **IPI00111183**, Tax_Id=10090 Gene_Symbol=Npr1 Atrial natriuretic peptide receptor A

Match to Query 600: 1263.537724 from(632.776138,2+)
Title: orbitrap002311.1180.1180.2.dta
Data file C:\Dokumente und Einstellungen\Juliane\Eigene Dateien\Orbitrap-Files\orbitrap002311.RAW.mgf

Click mouse within plot area to zoom in by factor of two about that point
Or, Plot from 150 to 1150 Da Full range

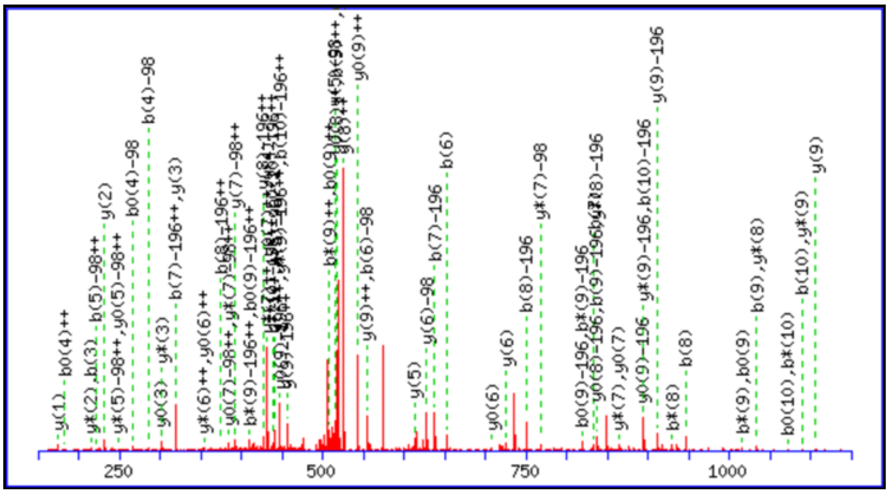

Monoisotopic mass of neutral peptide Mr(calc): 1263.5374
Fixed modifications: Carbamidomethyl (C)
Variable modifications:
S4 : Phospho (ST), with neutral losses 97.9769(shown in table), 0.0000
T7 : Phospho (ST), with neutral losses 97.9769(shown in table), 0.0000
Ions Score: 33 **Expect:** 0.017
Matches (Bold Red): 75/184 fragment ions using 101 most intense peaks

#	b	b^{++}	b*	b^{*++}	b^{0}	b^{0++}	Seq.	y	y^{++}	y*	y^{*++}	y^{0}	y^{0++}	#
1	88.0393	44.5233			70.0287	35.5180	S							11
2	159.0764	80.0418			141.0659	71.0366	A	981.5588	491.2830	964.5323	482.7698	963.5483	482.2778	10
3	**216.0979**	108.5526			198.0873	99.5473	G	**910.5217**	**455.7645**	**893.4952**	**447.2512**	**892.5111**	**446.7592**	9
4	**285.1193**	143.0633			**267.1088**	134.0580	S	853.5002	**427.2538**	**836.4737**	418.7405	**835.4897**	418.2485	8
5	**441.2204**	**221.1139**	424.1939	212.6006	423.2099	212.1086	R	784.4788	**392.7430**	**767.4522**	**384.2298**	766.4682	**383.7378**	7
6	**554.3045**	277.6559	537.2780	269.1426	536.2939	268.6506	L	**628.3777**	314.6925	611.3511	306.1792	610.3671	305.6872	6
7	**637.3416**	**319.1744**	620.3151	310.6612	619.3311	310.1692	T	**515.2936**	258.1504	498.2671	**249.6372**	497.2831	**249.1452**	5
8	**750.4257**	**375.7165**	733.3991	367.2032	732.4151	366.7112	L	**432.2565**	216.6319	415.2300	208.1186	414.2459	207.6266	4
9	**837.4577**	419.2325	**820.4312**	**410.7192**	**819.4471**	**410.2272**	S	**319.1724**	160.0899	**302.1459**	151.5766	**301.1619**	151.0846	3
10	**894.4792**	**447.7432**	877.4526	**439.2300**	876.4686	**438.7379**	G	**232.1404**	116.5738	**215.1139**	108.0606			2
11							R	**175.1190**	88.0631	158.0924	79.5498			1

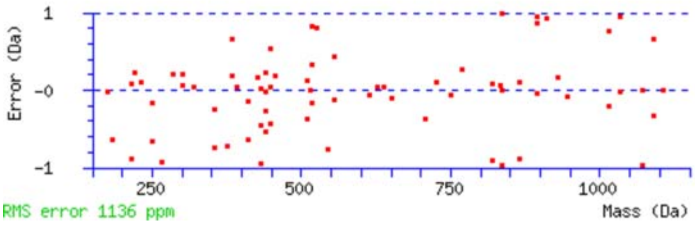

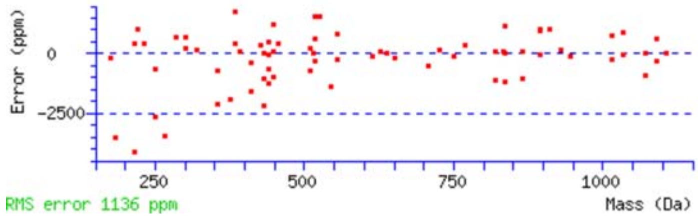

NCBI **BLAST** search of SAGSRLTLSGR
(Parameters: blastp, nr protein database, expect=20000, no filter, PAM30)
Other BLAST web gateways

All matches to this query

Score	Mr(calc):	Delta	Sequence
32.8	1263.5374	0.0004	SAGSRLTLSGR
30.2	1263.5374	0.0004	SAGSRLTLSGR
25.0	1263.5374	0.0004	SAGSRLTLSGR
22.5	1263.5374	0.0004	SAGSRLTLSGR
22.3	1263.5374	0.0004	SAGSRLTLSGR
16.6	1263.5374	0.0004	SAGSRLTLSGR
10.5	1262.5309	1.0069	LSLTEEKQR
9.5	1263.5357	0.0020	APGGSRASPGDGR
9.0	1262.5309	1.0068	TLVLQTSEGR
8.8	1263.5373	0.0004	XKKSAQSSVR

Mascot: http://www.matrixscience.com/

Mascot Search Results

Peptide View

MS/MS Fragmentation of **TYWLLGER**
Found in **IPI00111183**, Tax_Id=10090 Gene_Symbol=Npr1 Atrial natriuretic peptide receptor A

Match to Query 385: 1116.503724 from(559.259138,2+)
Title: orbitrap002311.2082.2082.2.dta
Data file C:\Dokumente und Einstellungen\Juliane\Eigene Dateien\Orbitrap-Files\orbitrap002311.RAW.mgf

Click mouse within plot area to zoom in by factor of two about that point
Or, Plot from 150 to 1050 Da Full range

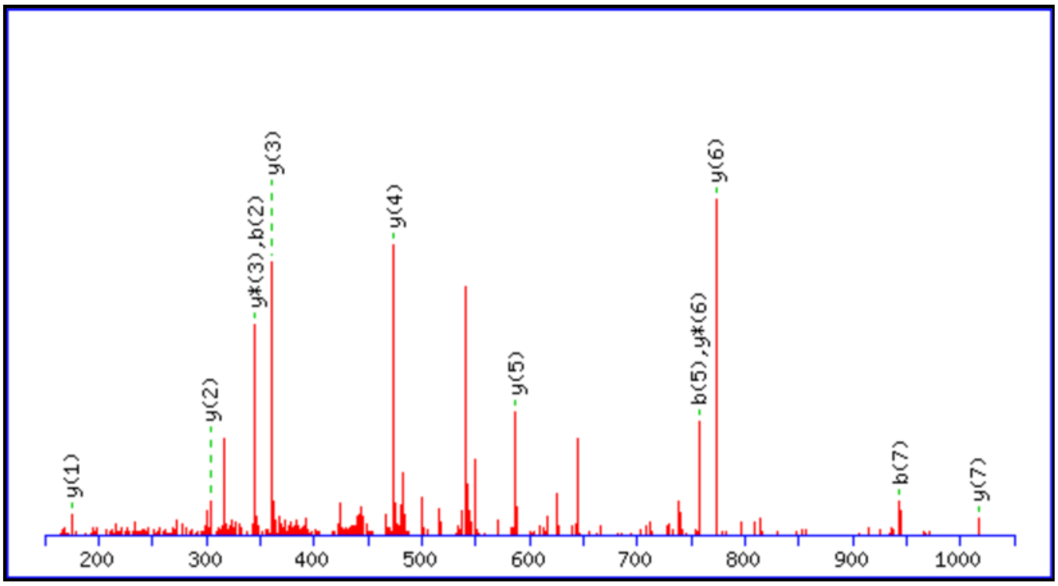

Monoisotopic mass of neutral peptide Mr(calc): 1116.5005
Fixed modifications: Carbamidomethyl (C)
Variable modifications:
Y2 : Phospho (Y)
Ions Score: 54 **Expect:** 0.0001
Matches (Bold Red): 12/68 fragment ions using 18 most intense peaks

#	b	b^{++}	b^0	b^{0++}	Seq.	y	y^{++}	y*	y^{*++}	y^0	y^{0++}	#
1	102.0550	51.5311	84.0444	42.5258	T							8
2	**345.0846**	173.0459	327.0740	164.0407	Y	**1016.4601**	508.7337	999.4336	500.2204	998.4495	499.7284	7
3	531.1639	266.0856	513.1534	257.0803	W	**773.4305**	387.2189	**756.4039**	378.7056	755.4199	378.2136	6
4	644.2480	322.6276	626.2374	313.6223	L	**587.3511**	294.1792	570.3246	285.6659	569.3406	285.1739	5
5	**757.3320**	379.1697	739.3215	370.1644	L	**474.2671**	237.6372	457.2405	229.1239	456.2565	228.6319	4
6	814.3535	407.6804	796.3429	398.6751	G	**361.1830**	181.0951	**344.1565**	172.5819	343.1724	172.0899	3
7	**943.3961**	472.2017	925.3855	463.1964	E	**304.1615**	152.5844	287.1350	144.0711	286.1510	143.5791	2
8					R	**175.1190**	88.0631	158.0924	79.5498			1

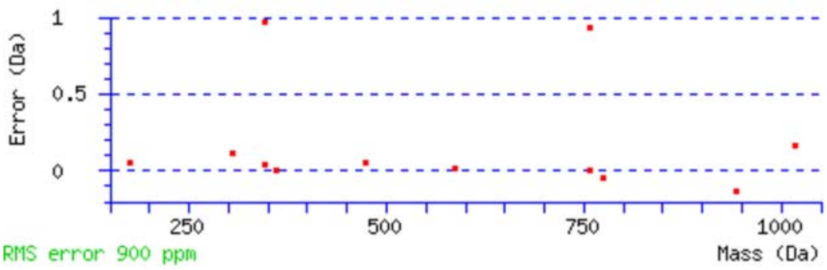

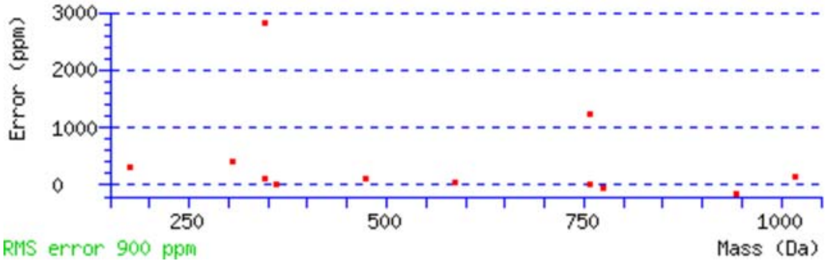

NCBI **BLAST** search of TYWLLGER
(Parameters: blastp, nr protein database, expect=20000, no filter, PAM30)
Other BLAST web gateways

All matches to this query

Score	Mr(calc):	Delta	Sequence
54.0	1116.5005	0.0032	TYWLLGER
38.0	1116.5005	0.0032	TYWLLGER
12.9	1115.5012	1.0025	SKEFALADR
10.9	1115.5012	1.0025	VSGVGIDYAR
8.3	1116.5077	-0.0040	ATNYGSRLR
8.1	1115.5012	1.0025	VQDLFTSAR
8.1	1115.5012	1.0025	VQDLFTSAR
5.7	1115.5043	0.9994	LCAEHGHHR
5.3	1115.5012	1.0025	AGPIFTSTSR
4.4	1116.5087	-0.0050	EDTGYYTLR

Mascot: http://www.matrixscience.com/
