# Supplementary material for: Homologous desensitization of guanylyl cyclase A, the receptor for atrial natriuretic peptide, is associated with a complex phosphorylation pattern
Source: FEBS J. 2010 Jun;277(11):2440–53. doi: 10.1111/j.1742-4658.2010.07658.x (PMC2901513; doi:10.1111/j.1742-4658.2010.07658.x)
Supplement: Supplementary file 4 [file ejb0277-2440-SD4.pdf]

**Supplementary Table S1:** Site-directed mutagenesis. Oligonucleotides and PCR reactions used to generate the plasmid for expression of GC-A S487E.

| Number | Name | Sequence | Tm [°C] |
|---|---|---|---|
| 1 | S487A/E fwd I | ctg gcc tct ggg ata tcc acc tc | 66.0°C |
| 4 | S487A/E rev II | ctt agg gca atc tcc tgc agg atg | 64.4°C |
| 5 | S487E fwd II | gag tta gaa cgc cac tta cgg agc gct ggc agc cgg ctg acc | 80.2°C |
| 6 | S487E rev I | gtg gcg ttc taa ctc gct ggg ctg caa gtc ctc cca gcg cac | 80.2°C |

**Legend to supplemental Table S1:**

The wild type FLAG-tagged GC-A from rat in pCMV5 expression vector was used as a template for PCR mediated mutagenesis of serine 487 to glutamate. Two independent PCRs (PCR I: primer 1 + primer 4; PCR II: primer 2 + primer 3) were set up at an annealing temperature of 55 °C using 5 ng of template DNA, a final primer concentration of 20 pM and 1 unit of Taq DNA Polymerase (Invitrogen, Karlsruhe, Germany). PCR products were separated by gel electrophoresis and extracted using a commercial available kit (Macherey-Nagel, Düren, Germany). The products of PCRI ( ~ 250bp) and PCRII ( ~ 650bp) were fused to generate a DNA strain carrying the S487E mutation. Briefly, a 20 µl PCR reaction was set up with 1 µl of each PCR product, 1 unit of Taq DNA polymerase and 2 nM dNTP were annealed at 60°C. After 10 cycles reaction was cooled down to 4°C, 20 pM of primer 1 and primer 2 were added and the reaction was continued for 25 cycles. Q-Solution (Qiagen, Hilden, Germany) was added according to the manufacturer to all PCR reactions to improve the specificity. Fused PCR products (879bp) were purified by Chloroform/Isoamyl alcohol extraction. The pCMV5-FLAG-GC-A plasmid and the purified DNA strain carrying the expected mutation were subjected to double digestion with 5 units of EcoRV and SbfI (New England Biolabs, Frankfurt/Main, Germany) and ligated with the mutated strain using T4 DNA Ligase (New England Biolabs, Frankfurt/Main, Germany). Resulting constructs were verified through sequencing using internal primers.
